# Fluorescence Properties and Mg^2+^ Selectivity of Aryl‐Alkynyl Derivatives of the *o*‐Aminophenoltriacetate (APTRA) Ligand

**DOI:** 10.1002/chem.202503428

**Published:** 2026-01-07

**Authors:** Laura L. Duncan, Christopher Hogg, J. A. Gareth Williams

**Affiliations:** ^1^ Department of Chemistry Durham University Durham UK

## Abstract

*Ortho*‐aminophenol‐*N,N,O*‐triacetate (APTRA) has been widely adopted for binding and sensing Mg^2+^, although it binds Ca^2+^ more strongly. This contribution investigates APTRA derivatives that incorporate an aryl‐alkynyl fluorophore, addressing how the binding affinities for Mg^2+^ and Ca^2+^ are modulated by a substituent *R* in the aryl ring of the fluorophore. Six such derivatives have been synthesized via their *tris*‐ethyl esters. They feature *X* ═ CN, CF_3_, or OMe, as a mesomerically electron‐withdrawing, inductively electron‐withdrawing, or electron‐donating substituent, respectively, with the alkyne either *para* or *meta* to the APTRA nitrogen. Study of the absorption and fluorescence properties of the esters reveals the importance of intramolecular charge transfer (ICT) states for *X* ═ CN and CF_3_, but not OMe. The corresponding carboxylate ligands are less emissive in water, and the fluorescence is not strongly modulated by metal ions. However, the absorption spectra change markedly, allowing dissociation constants *K*_d_ to be evaluated. The key conclusions are that (i) electron‐withdrawing substituents attenuate the affinity for Ca^2+^ more than Mg^2+^, leading to a net improvement in selectivity for Mg^2+^, and (ii) the effect is larger when the APTRA nitrogen (as opposed to phenolic oxygen) is *para* to—and hence directly conjugated with—the alkyne.

## Graphical abstract

It is a challenge to improve the selectivity of high‐denticity ligands for Mg^2+^ over Ca^2+^. We show that in aryl‐alkynyl derivatives of APTRA, electron‐withdrawing substituents lower the binding affinity for Ca^2+^ more than Mg^2+^, making the ligand more selective for Mg^2+^. The effect is larger when the APTRA nitrogen is *para* to alkyne, but the associated charge‐transfer fluorescence is weak in water.

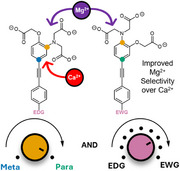

## 1 Introduction

In mammalian cells, the magnesium ion Mg^2+^ is the second most abundant cation after K^+^, with the concentration of the “free” ion typically in the range 0.5 to 1.8 mM [1, 2, 3, 4] (“free” Mg^2+^ here refers to the hydrated cation, with no other endogenous ligands coordinated). Mg^2+^ has numerous crucial roles in cells ranging from the stabilization of nucleic acids through to the catalysis of protein synthesis; indeed, it is a cofactor in at least 600 known enzymatic reactions. Nevertheless, the intracellular dynamics of Mg^2+^ remain largely unknown, primarily due to the inability to detect and monitor Mg^2+^ reliably in vivo. This contrasts with the deeper understanding of ions like Ca^2+^ and Zn^2+^, for example, where the successful development of selective and sensitive fluorescent probes has allowed them to be studied routinely using fluorescence microscopy [5, 6, 7].

**FIGURE 1:**
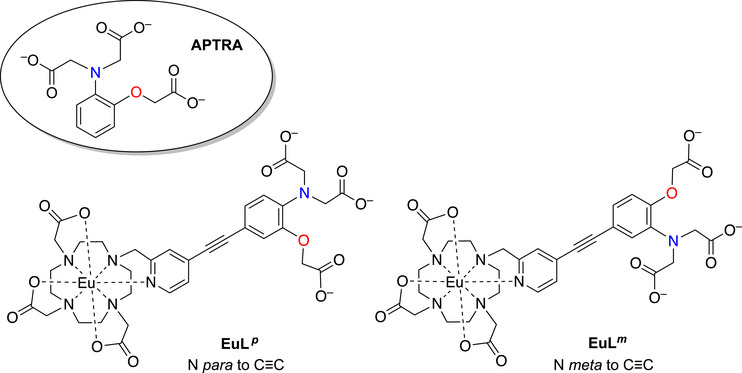
The structure of APTRA (circled) and the two previously reported probes that incorporate an APTRA unit linked to a europium‐based emitter through an alkyne, either *para* or *meta* to the APTRA nitrogen shown in blue (EuL ^*p*^ and EuL^*m*^ respectively) [38].

The sensitivity coupled with the spatial and temporal resolution of fluorescence microscopy render it an attractive technique for such studies, but it requires molecules that bind *selectively* to the target ion. There are few such molecules that do so well for Mg^2+^. The most widely used is *o*‐aminophenol‐*N*,*N*,*O*‐triacetate (APTRA, circled structure in Figure 1) [8, 9, 10]. It is a pentadentate ligand that was developed from the octadentate BAPTA, used so successfully for Ca^2+^ {BAPTA = 1,2‐bis(*o*‐amino‐phenoxy)ethanetetraacetic acid} [11, 12]. The lower denticity helps to disfavor the binding of the larger Ca^2+^ ion compared to Mg^2+^. Nevertheless, Ca^2+^ and Zn^2+^ do coordinate to APTRA competitively with Mg^2+^ (binding constants are summarized in Table 1), and interference from these ions is observed for most of the reported APTRA‐derived luminescent probes in which an emitting unit is linked to the phenyl ring of APTRA [13, 14, 15, 16, 17, 18, 19, 20]. Alternative binding groups have also been investigated, including bidentate [21, 22, 23, 24, 25, 26] and tridentate [27] binding units where the lower denticity helps to favor the binding of the smaller Mg^2+^ ion [28, 29]. However, these probes are more prone to forming ternary complexes with polyphosphates in vivo, such that they cannot necessarily detect free Mg^2+^ accurately [3]. Recently described amine‐appended HNBO fluorophores {HNBO = 2‐(2‐hydroxy‐3‐naphthyl‐4‐methyl‐benzoxaole)} have shown promising Mg^2+^ selectivity in mixed organic–aqueous media or on solid supports, though not hitherto in fully aqueous solution [30, 31]. Despite such progress, therefore, APTRA‐based systems remain of much interest [7, 8, 9, 10, 11, 12, 13, 14], as do promising new APTRA analogues in which one of the carboxylates is replaced by a phosphinate [32, 33, 34], or the phenolic oxygen by a sulfur [35].

**TABLE 1:** Dissociation constants (*K*_d_) for APTRA, EuL ^*p*^, and EuL^*m*^.

Ligand	*K*_d_ Mg^2+^	*K*_d_ Ca^2+^	*K*_d_ Zn^2+^
**APTRA** ^a^	1.9 ± 0.1 mM	9.8 ± 0.7 µM	14 ± 3 nM
**EuL *^p^*** ^b^	7.5 ± 0.8 mM	900 ± 200 µM	1800 ± 500 nM
**EuL*^m^*** ^b^	3.7 ± 1.5 mM	900 ± 100 µM	1900 ± 100 nM

^a^ Data from the *K*_a_ values (where *K*_d_ = *K*_a_^−1^) in ref. [9], in PIPES‐buffered aqueous solution, pH 7.0, 0.1 M KCl, 298 K.

^b^ Data from ref. 28, in HEPES‐buffered aqueous solution, pH 7.2, 0.1 M KCl, 298 K.

Aside from applications in the *detection* of Mg^2+^, there is equally a growing need, in cell biology and physiology research, for cell‐permeable tailor‐made ligands that can *perturb* metal ion concentrations and bioavailability selectively. For example, recent studies have highlighted the importance of divalent metal ions—particularly Mg^2+^ and Ca^2+^—in determining the formation and structure of chromatin [36, 37]. Access to ligands with different selectivities for these metal ions has offered deeper insight and may ultimately reveal how metal ion flux influences gene expression, for instance [37]. In such applications, no fluorescent reporter group is required: it is the ligating unit alone that is crucial, and hence also the ability to control relative metal ion affinities and selectivity.

Previously, we unexpectedly discovered that the conjugation of APTRA to a lanthanide‐based emitting unit through an acetylide linker led to systems EuL *^p^* and EuL*^m^* (Figure 1) that have much higher selectivity for Mg^2+^ than does APTRA itself [38]. The dissociation constants (Table 1) showed how this improvement in selectivity arises from the much larger reduction in affinity for Ca^2+^ and Zn^2+^ than Mg^2+^. Yet, the underlying reasons for this trend remain unclear. Electronic factors or differences in solvation (or, indeed, a combination of both) could be responsible. In terms of electronics, we noted that the pyridyl unit bound to the Eu^3+^ ion could act as a strong charge sink [38, 39], diminishing the availability of the anilino N and phenolic O lone pairs in the APTRA unit for binding. This lone pair conjugation might be anticipated to disfavor the binding of Ca^2+^ more significantly than Mg^2+^. The position of these atoms relative to the alkyne (i.e., N or O *para* to C≡C) has a small effect on Mg^2+^ affinity but also determines the nature of the fluorescence change: a partial “turn‐off” but ratiometric (in excitation) response for EuL *^p^*, compared to a nonratiometric “turn‐on” response for EuL*^m^*. Again, the origin of the difference is unclear but electronic factors must be involved.

**FIGURE 2:**
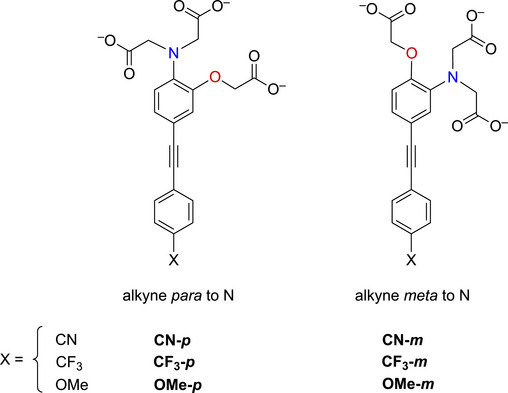
The structures of the six new aryl‐alkynyl‐APTRA derivatives synthesized and studied in this work.

The unexpectedly large effect of the emitting unit in these systems on the relative affinities for the different metal ions led us to scrutinize existing literature for examples of electronic effects of substituents influencing selectivity, but there appear to be no systematic studies. To probe further how electronic factors associated with the fluorophore may influence the metal binding and response in aryl‐alkynyl‐appended probes, we deemed it desirable to study examples of purely organic systems in which the electron‐withdrawing or ‑donating nature of the aryl group is varied. The only other two such examples reported to date feature an alkynyl‐1‐naphthyl group *para* or *meta* to the APTRA nitrogen, but they shed no light on the question, as the naphthyl substituent is common to both [19]. In the present work, we therefore set out to prepare a series of six new such compounds (Figure 2) and study the changes in their absorption and fluorescence spectra induced by Mg^2+^ and Ca^2+^. The compounds feature *para*‐substituted alkynyl arenes where the substituent is (i) mesomerically electron‐withdrawing (C≡N), or (ii) inductively electron‐withdrawing (CF_3_), or (iii) electron‐donating (OMe). For each substituent, the pair of isomeric molecules have been studied in which the alkyne is positioned either *para* or *meta* to the alkyne. The compounds will accordingly be referred to by the substituent followed by *p* or *m* (Figure 2). Moreover, despite existing literature on the excited states of a few specific donor–acceptor diphenylacetylene derivatives (vide infra), there is no systematic study on how other substituents (e.g., donor–donor) influence the fluorescence. We have therefore also evaluated the absorption and emission properties of the ester precursors to the six ligands, in order to explore the effect of substituent pairings.

## 2 Results and Discussion

### 2.1 Synthesis

**SCHEME 1:**
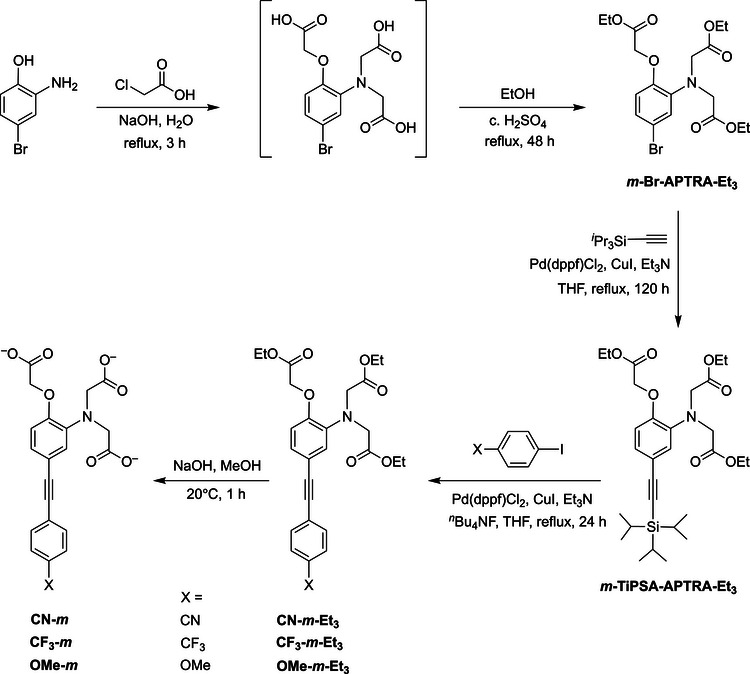
Synthetic route to the *meta*‐linked family of compounds CN‐*m*, CF_3_‐*m*, and OMe‐*m*. The *para*‐linked isomers were prepared similarly from *p*‐TiPSA‐APTRA (Scheme S1).

The six new APTRA‐based probes were synthesized as their ethyl esters by one‐pot deprotection and palladium‐catalyzed Sonogashira cross‐coupling of the corresponding TiPS (tri‐isopropylsilyl)‐protected *meta‐* or *para*‐alkynyl‐functionalized APTRA esters with the appropriate *para*‐substituted iodobenzene (Scheme 1 and Scheme S1, respectively; details of experimental procedures and characterization of new compounds are provided in the Experimental section and Supporting Information).

The cross‐couplings proceeded satisfactorily in around 24 h in THF in the presence of triethylamine, using Pd(dppf)Cl_2_ and CuI as the catalyst, and with *^n^* Bu_4_NF added to remove the silyl group in situ. The synthesis of the requisite *m*‐ and *p*‐TiPSA‐APTRA‐Et_3_ intermediates was likewise accomplished by Sonogashira coupling of *m*‐ and *p*‐Br‐APTRA‐Et_3_ with TiPS‐acetylene. Contrary to our previous work, in which *m*‑Br‑APTRA‐Et_3_ was prepared by alkylation of 2‐amino‐4‐bromophenol with ethylbromoacetate over a period of 5 days, we found that this compound could be prepared more quickly and economically by alkylation of the starting material with chloroacetic acid in aqueous sodium hydroxide, followed by acid‐catalyzed esterification in ethanol [40, 41]. The six ester‐protected probes were hydrolyzed using base in methanol to give the carboxylates (i.e., the free ligands, as opposed to the ester proligands), as and when required for study (Schemes 1 and S1). (Note that, as some APTRA derivatives have been reported to be somewhat unstable as the free ligands in aqueous solution [9, 18], the new probes were stored as the ethyl esters until required). The electron‐donating or withdrawing nature of the X substituents is reflected in the ^1^H and ^13^C NMR spectra, most notably by the C and H *ortho* to the substituent, as expected. The ^1^H resonance is shifted from the range 7.5 – 7.7 ppm for X = CN and CF_3_ to the range 6.8 – 7.0 ppm for X = OMe; likewise, the ^13^C shifts from around 132 to 114 ppm. For a given substituent, the NMR resonances within the ring show little variation between the *p* and *m* series, or between the esters and the carboxylates.

### 2.2 Absorption and Emission Properties

#### 2.2.1 Literature Precedent

**FIGURE 3:**
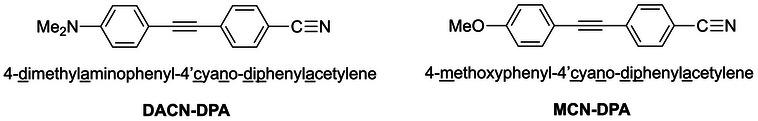
The structures of DACN‐DPA and MCN‐DPA, of which the new compounds CN‐*p* and CN‑*m*, respectively, may be considered derivatives.

The new family of compounds prepared are derivatives of diphenylacetylene (DPA). The excited‐state properties and photophysics of DPA itself have been extensively studied [42, 43, 44]. Donor–acceptor (D−A) derivatives of DPA—notably 4‐dimethylamino‐4**′**‐cyanodiphenylacetylene [45, 46, 47] (DACN‐DPA), and 4‐methoxy‐4**′**‐cyanodiphenylacetylene [48, 49, 50] (MCN‐DPA) (Figure 3)—have been investigated in the context of intramolecular charge‐transfer (ICT) excited states and nonlinear optical properties (albeit to a lesser extent than the corresponding C═C stilbenes [51]). The CN‐*p* compound in the present study would be expected to display properties similar to DACN‐DPA, perhaps perturbed marginally by the additional phenolic substituent *meta* to the C≡C. On the other hand, there is essentially no precedent for the excited‐state properties of the CF_3_ or OMe derivatives (weaker, inductive‐only acceptor, and weaker donor respectively), neither *p* nor *m*. Moreover, previous studies on DPAs are almost exclusively limited to organic solvents: the derivatives examined have not been water‐soluble. Before assessing the metal‐binding properties of the new ligands, therefore, we deemed it important to study the absorption and emission properties of the family of six precursor esters in an organic solvent, as well as the six hydrolyzed compounds in water.

A combination of experimental and theoretical studies have revealed a complex excited‐state landscape for DACN‐DPA, with five excited singlet states of similar energies: two “localized” states, and three ICT states, with different conformations of the rings relative to one another or with the Me_2_N either in‐plane or twisted relative to the phenyl ring to which it is attached [47]. The key point though, as far as steady‐state properties are concerned, is that increasingly red‐shifted emission is observed as solvent polarity increases, as the lowest‐energy ICT state becomes increasingly stabilized, reminiscent of classic “TICT”‐forming compounds like *p*‐dimethylamino‐benzonitrile (TICT = twisted intramolecular charge transfer excited state). MCN‐DPA likewise emits from an ICT state in polar solvents [48, 49, 50]. As noted above, however, studies of such molecules have been limited to organic solvents, the most polar typically being MeCN or *n*‐BuOH.

### 2.3 Properties of the Ethyl Esters

**FIGURE 4:**
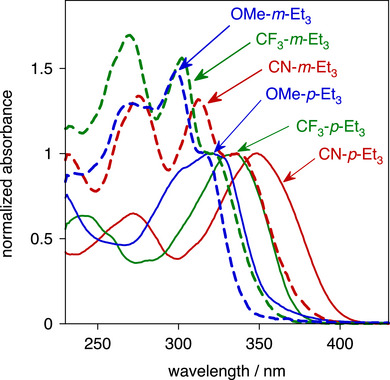
Absorption spectra of the esters in dichloromethane at 295 K, normalized at the longest‐wavelength absorption band. The ‘*p*’ family are represented by thin solid lines, and their ‘*m*’ isomers by thicker dashed lines; substituents are colored red = CN; green = CF_3_; blue = OMe.

The normalized UV‐visible absorption spectra of the six esters in dichloromethane solution are shown in Figure 4, and numerical data including extinction coefficients are listed in Table 2. The spectra of the *p* family at *λ* > 250 nm are dominated by one main, broad band, whereas the *m* isomers show three bands over this region. Within each family, the energy of the longest‐wavelength absorption band decreases in the order OMe > CF_3_> CN. This is in line with the increasingly electron‐accepting nature of the substituents (Hammett *σ*_p_ parameters are −0.27, 0.54, and 0.66 respectively [52, 53]), complementing the electron‐donating amino and phenolate groups of the APTRA ring. The enhanced D–A character leads to an effectively more extended conjugated system and to lower energy absorption. Likewise, for each of the three substituents, the *p* compound (with the amine *para* to C≡C) absorbs at lower energy than its *m* isomer (amine N *meta* to C≡C, oxygen *para*), which can similarly be accounted for in terms of the more strongly electron‐donating nature of the amine enhancing the D−A character when *para*. Accordingly, the difference in energy within each pair becomes smaller as the electron‐accepting nature of the substituent decreases: Δ*Ε*_m–p_ = 2100, 1400, 300 cm^–^^1^ for CN, CF_3_, OMe, respectively.

**TABLE 2:** Absorption and emission data for the six precursor esters in CH_2_Cl_2_ at 295 K.

		Emission				
Ester	Absorption^a^ *λ*_max_ [nm] (ε / M^−1^ cm^−1^)	*λ*_max_ [nm]	*Φ*_f_ ^b^	*τ* [ns]^c^	*k*_r_/ 10^8^ s^−1^ ^d^	∑*k*_nr_/ 10^8^ s^−1^ ^d^
**CN‐*p***	272 (15300), 349 (23900)	470	0.37	1.5	2.5	4.2
**CF_3_‐*p***	241 (15900), 259 sh (12700), 335 (25000)	425	0.27	2.0	1.4	3.7
**OMe‐*p***	302sh (17200), 321 (18900)	363	0.01	3.6	0.028	2.8
**CN‐*m***	276 (26600), 312 (26200), 335 (19800)	475	0.56	5.5	1.0	0.80
**CF_3_‐*m***	269 (26400), 302 (24400), 322 sh (15600)	427	0.44	3.7	1.2	1.5
**OMe‐*m***	272 (26000), 298 (29800), 315 sh (20100)	368	0.03	7.0	0.043	1.4

^a^ Bands centered at λ > 240 nm are listed; sh = shoulder.

^b^ Fluorescence quantum yield measured using quinine sulfate in 1 M aqueous sulfuric acid as the standard.

^c^ Fluorescence lifetime measured by time‐correlated single photon counting (TCSPC), λ_ex_ = 374 nm.

^d^ *k*_r_ and ∑*k*_nr_ are the rate constants of radiative and nonradiative decay, respectively, estimated assuming that the emissive state is formed with unit efficiency, such that *k*_r_ = *Φ* / *τ* and ∑*k*_nr_ = (1 – *Φ*) / *τ*.

**FIGURE 5:**
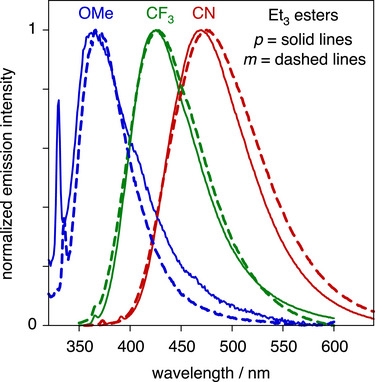
Normalized emission spectra of the esters in dichloromethane at 295 K; line style and color coding as in Figure 4; *λ*_ex_ / nm = 300 (*p*‐OMe), 305 (*m*‐OMe), 330 (*m*‐ and *p*‐CF_3_), 335 (*m*‐CN), and 350 (*p*‐CN). (The sharp bands at higher energy for the OMe derivatives are C─H Raman of the solvent; they become conspicuous as the emission of these two derivatives is weak).

The photoluminescence spectra of the esters in dichloromethane solution at 295 K (Figure 5) each display a single, broad band. The emission energy (based on *λ*_max_) decreases unequivocally in the order OMe > CF_3_ > CN, consistent with the trend in absorption and with the more electron‐withdrawing substituents stabilizing the emissive state. Within each pair of compounds, however, the emission energy is now almost independent of whether the amine is *para* or *meta* to the C≡C. This contrasts with the absorption trend and may at first appear counterintuitive. It is, however, probably consistent with what would be expected if the emission emanates from a twisted ICT state of the type inferred for DACN‐DPA in polar aprotic solvent by Lim and colleagues [47]. They attributed the emission of this parent compound in acetonitrile to a twisted “ICT(σ*)” state, involving transient transfer of an electron from the Me_2_N─C_6_H_4_─C≡C─ unit to the ─C_6_H_4_─C≡N. Computations revealed an excited‐state geometry in which the plane of the former (the electron donor, D) is perpendicular to the latter (the acceptor), and with an angle of 135° for D─Ĉ≡C, as opposed to linear in the ground S_0_ and initially populated S_1_ (π‐π*) states. The twisting in the excited state thus decouples the two units, and so the emission energy of molecules forming such states should be essentially independent of the position of the amine donor within the ring, but dependent only on the through‐space donating power of the aniline unit.

Whilst such an explanation likely applies to the CN derivatives (for which DACN‐DPA should be a good model), and probably also to the CF_3_ derivatives (featuring a good electron‐acceptor), it seems unlikely that the OMe pair of compounds will generate ICT states at all, as the ─C_6_H_4_─OMe unit is not electron‐accepting. Support for a different nature to the excited states of the OMe compounds is provided by the trend in quantum yields. Thus, the CN‐ and CF_3_‐substituted compounds are bright emitters with fluorescence quantum yields *Φ*_f_ in the range 27% to 56% and lifetimes *τ* in the nanosecond range, but the OMe compounds are only weakly emissive (Table 2). Assuming that the emissive state is formed with approximately unit efficiency, the rate constants for radiative and nonradiative decay (*k*_r_ and ∑*k*_nr_ respectively) can be estimated from the *Φ*_f_ and *τ* values. The values so obtained (Table 2) indicate that the suppressed emission in the methoxy derivatives is due to the *k*_r_ values being one to two orders of magnitude lower than for the other compounds, suggesting a different orbital parentage for the emissive state of the OMe pair. On the other hand, the lowest‐energy absorption bands of these two compounds have molar absorptivities comparable to those of the other compounds, implying similar oscillator strengths for transitions from the ground to the initially formed excited state (expected to be the localized ^1^π‐π* state). It thus seems likely that for the OMe compounds, the initially formed ^1^π‐π* excited state—instead of then populating an ICT state like the other compounds with greater D–A character—leads to a π‐σ* state of the type identified by Lim and colleagues as a higher state for DACN‐DPA [47]. This is a planar state involving the promotion of an electron to the σ* orbital on the acetylenic bond, and its lower dipole moment may account for the suppressed *k*_r_ and hence weaker emission.

### 2.4 Properties of the Carboxylates

**FIGURE 6:**
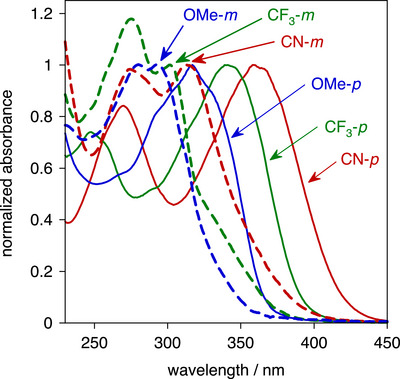
Absorption spectra of the carboxylates in buffered aqueous solution (pH = 7.2) at 295 K, normalized at the longest‐wavelength absorption band; line styles and color coding as in Figures 4, 5.

The normalized UV‐visible absorption spectra of the hydrolyzed compounds in water at pH 7.2 are shown in Figure 6. The spectra of the *p* family resemble those of the corresponding esters, being dominated by one broad band whose energy again decreases in the order OMe > CF_3_ > CN. For a given substituent, the *λ*_max_ values for the ester and carboxylate are similar (Table 3). The *m* family show two main bands, as opposed to three in the esters, although closer inspection reveals a shoulder on the long wavelength side that probably corresponds to the third (lowest‐energy) band in the esters. Again, for each of the three substituents, the *p* compound absorbs at lower energy than its *m* isomer, due to the D–A character effectively extending the conjugated system and lowering the π‐π* energy.

**TABLE 3:** Absorption and emission λ_max_ values for the carboxylate ligands (L) in water at 295 K and when saturated with Mg^2+^ or Ca^2+^. Data for the precursor esters **L‐Et_3_** from Table 2 are also reproduced here to aid comparison.

	Absorption *λ*_max_ [nm]			Emission *λ*_max_ [nm]			
Substituent	L‐Et_3_	L	L + M^2+ [^ ^a^ ^]^	L‐Et_3_	L	L + Mg^2+^	L + Ca^2+^
**CN‐*p***	349	362	320	470	551	539	503
**CF_3_‐*p***	335	341	311	425	488	477	479
**OMe‐*p***	321	330sh	315	363	432	385	378
**CN‐*m***	335	350sh	314	475	431	411	422
**CF_3_‐*m***	322sh	331sh	293	427	395	373	376
**OMe‐*m***	315sh	314sh	294	368	423	400	400

^a^Saturated with Mg^2+^ *or* Ca^2+^: the final values are the same for both metal ions within the uncertainty on *λ*_max_.

**FIGURE 7:**
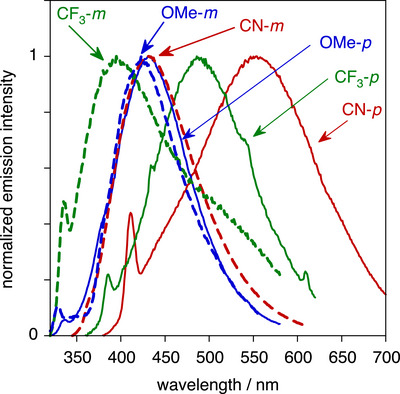
Normalized emission spectra of the carboxylates in buffered aqueous solution (pH = 7.2) at 295 K; line styles and color coding as in Figures 4, 5, 6; *λ*_max_ / nm = 295 (*m*‐OMe), 300 (*p*‐OMe and *m*‐CF_3_), 325 (*m*‐CN), 340 (*p*‐CF_3_), and 360 (*p*‐CN).

The fluorescence of the free carboxylates in water is much weaker than that of the esters in dichloromethane, to the extent that O–H Raman bands of water rather compromise the quality of the spectra (Figure 7). Nevertheless, all the compounds do show one emission band. For the *p* family, the emission energy varies significantly with the substituent, again decreasing in the order OMe > CF_3_ > CN. Strikingly, however, for the electron‐withdrawing CN and CF_3_ substituents, there is now a large difference between the emission *λ*_max_ for the *p* versus *m* isomers, the former being red‐shifted by over 4000 cm^−1^ while the latter emit at high energy. This contrasts with the esters, where *λ*_max_ is essentially invariant with position of the substituent, and it also contrasts with the pair of OMe compounds. The red‐shift is consistent with the charge‐transfer state of the CN and CF_3_ compounds being stabilized in the more polar aqueous solution; the expected larger change in dipole moment for the *p* series will likewise account for the greater stabilization of these compounds compared to the *m*. Within the *m* family, the substituent has a much lesser effect.

### 2.5 Effect of Metal Binding on the Absorption and Emission Spectra

**FIGURE 8:**
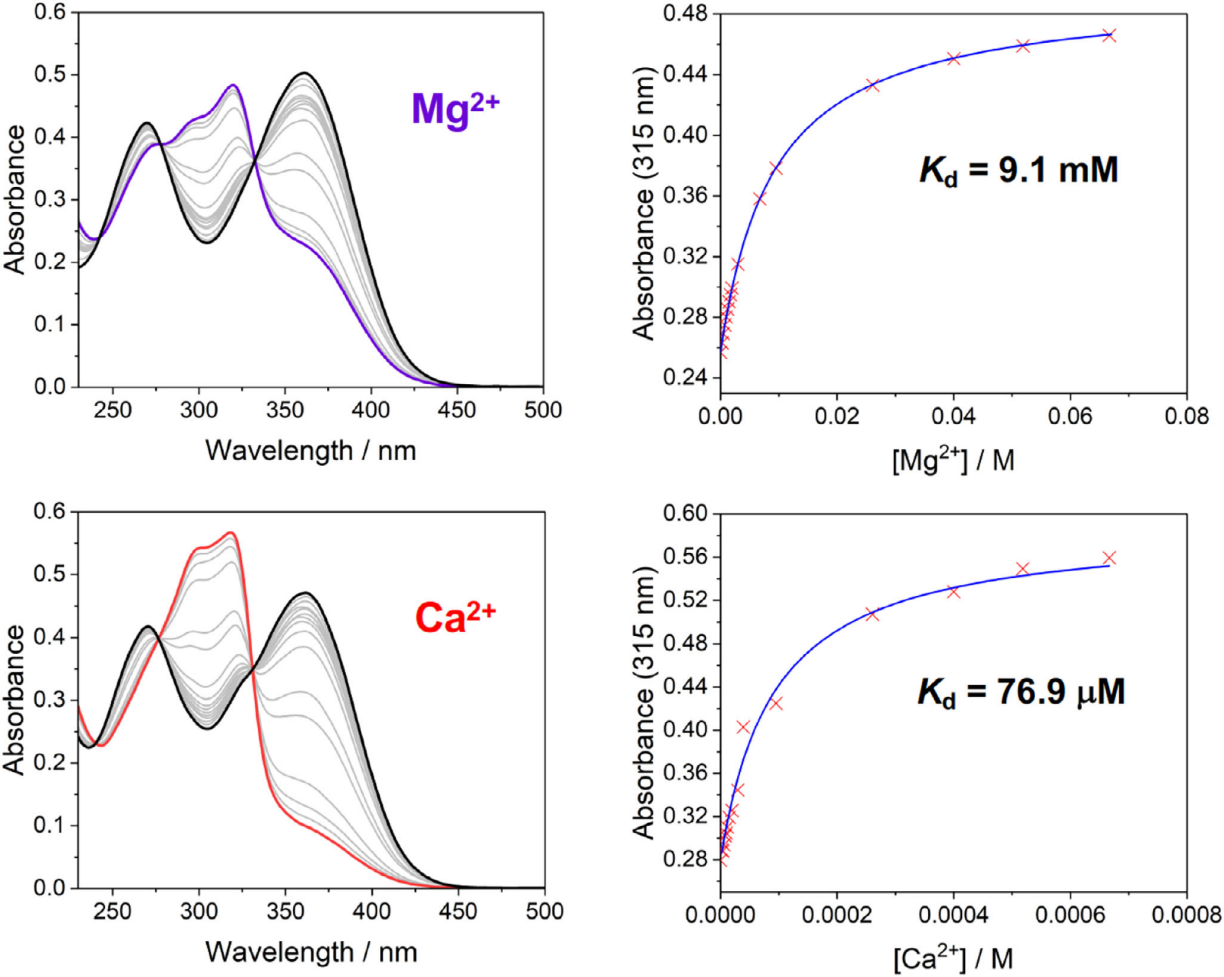
**Left**: The evolution of the absorption spectra of CN‐*p* with increasing amounts of Mg^2+^ (top) or Ca^2+^ (bottom); the spectrum of the free ligand is shown in black in each plot; spectra when saturated with Mg^2+^ and Ca^2+^ are in purple and red respectively; spectra at intermediate concentrations of the metal ions are in grey. **Right**: The change in absorbance at 315 nm as a function of [M^2+^]; data points are red crosses, with the fit in blue leading to the *K*_d_ values shown. [CN‐*p*] = 25 mM in aqueous buffer, 50 mM HEPES, pH 7.2, 0.1 M KCl, M^2+^ added as the chloride salt.

**FIGURE 9:**
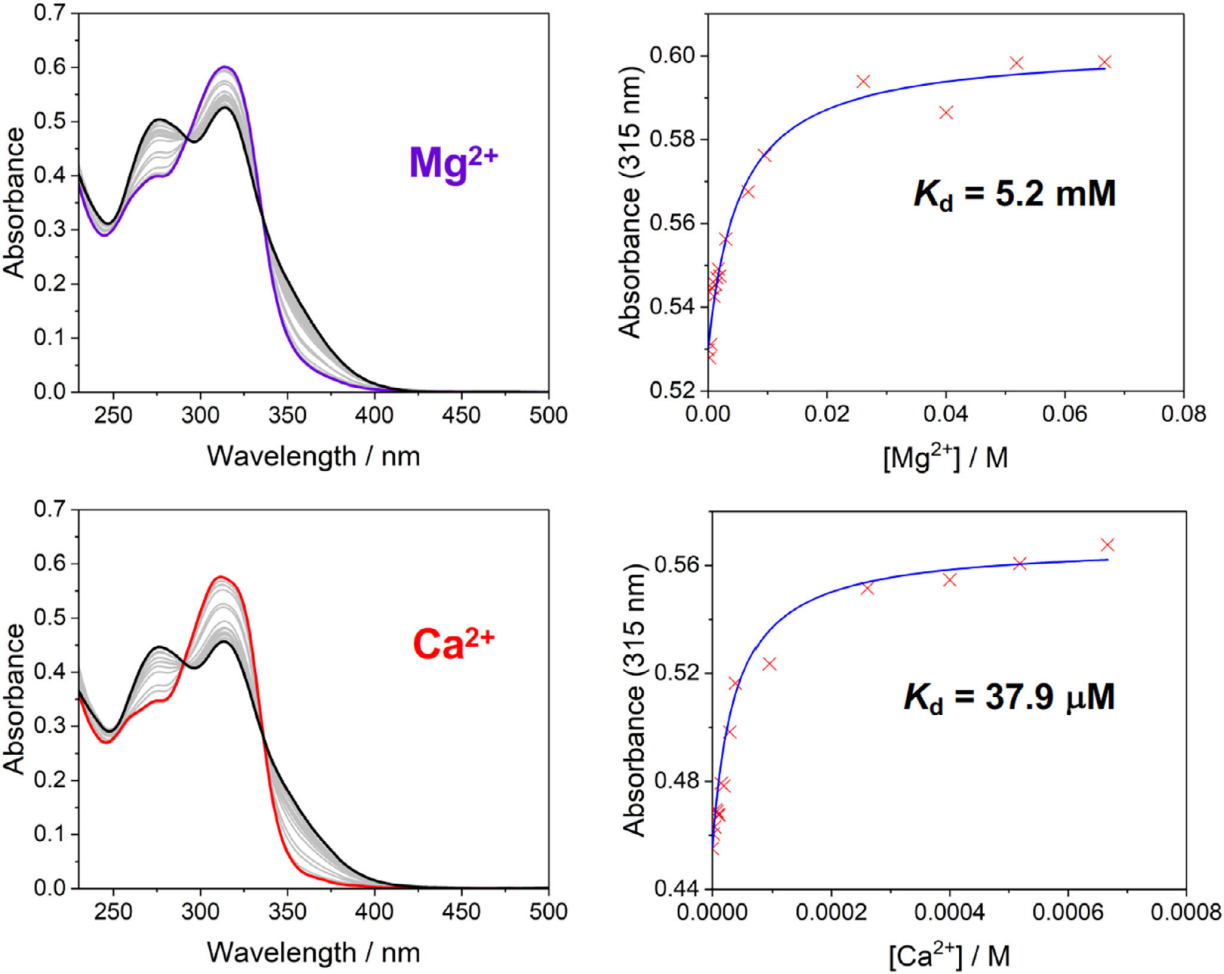
The evolution of the absorption spectra of CN‐*m* with Mg^2+^ and Ca^2+^; legend and conditions as for Figure 8.

The effect of binding of Mg^2+^ and Ca^2+^ on the absorption and emission spectra of the carboxylate ligands was probed by titrations with MgCl_2_ or CaCl_2_ in HEPES‐buffered aqueous solution (pH  =  7.2) at ambient temperature, in the presence of 0.1 M KCl. The limiting absorption and emission *λ*_max_ values at saturation (i.e., when no further changes in the spectra are detectable) are summarized in Table 3. The evolution of the absorption spectra with increasing concentrations of the metal ions is displayed in Figures 8 and 9 for CN‐*p* and CN‐*m* as representative examples; corresponding spectra for the other compounds are shown in Figures S1–S4. For CN‐*p* and CF_3_‐*p*, the spectral profiles change profoundly upon metal binding, with clear‐cut blue‐shifts and “new” bands emerging at shorter wavelength. The observation is consistent with the notion that binding of M^2+^ at the N (and O) atom will attenuate the donor character of the APTRA ring and thus raise the energy of the charge‐transfer state. The blue‐shift for OMe‐*p* is small, in line with minimal D–A character when the DPA unit is substituted with donor units at both ends. The absorption spectra of the *m* series do not show such a profound change, but the long wavelength shoulder is suppressed in the same manner when the metal binds.

The emission spectra likewise show blue shifts upon metal ion binding (Figures S5, S6 and Table 3), but the shift is only very small for the CN and CF_3_ ligands, likely due to the charge‐transfer excited state depleting the electron density at the APTRA nitrogen, thus reducing the affinity for metal ions. On the other hand, the OMe compounds, especially the *p* isomer, show a larger effect. This may at first appear at odds with the absorption results (where the shift was smaller for OMe than for the other substituents), but it is consistent with the earlier conclusion that these compounds do not form a charge‐transfer state. Thus, they likely retain higher affinity for M^2+^ ions in the excited states (of more localized character) from which they emit. Intensity changes are, of course, often used to signal a binding event, instead of wavelength shifts, even if they do not offer the advantage of ratiometric detection. For the *m* family of compounds, metal binding is accompanied by an increase in the intensity of fluorescence in each case (Figure S5), as had been found for *meta* isomer EuL*^m^* (Figure 1) [38]. This is probably largely due to the suppression of ∑*k*_nr_ as the binding of the metal enhances the rigidity of the fluorophore by restricting motion around the substituents. On the other hand, the intensity changes are smaller and more erratic for the *p* series, which may indicate that such suppression of nonradiative decay is counteracted by reduction in *k*_r_, with the binding of the metal having an expected, more profound influence on the character of the emissive state and suppressing the allowedness of the transition.

Given these generally rather small spectral responses and the weakness of the emission, coupled with the need for high‐energy excitation (the wavelengths employed are indicated in the caption to Figure 7) and the interference from solvent Raman bands, we focused on the use of the absorption spectra to evaluate and compare the metal binding constants of the six ligands. The absorbance at a suitable wavelength was monitored as a function of increasing [M^2+^] and the data were fitted to a 1:1 binding model, as shown in the right‐hand panels of Figures 8 and 9 for CN‐*p* and CN‐*m* (absorbance monitored at 315 nm), and in Figures S1–S4 for the other ligands (absorbance monitored at the wavelengths indicated in each figure). The 1:1 binding is the generally accepted stoichiometry for APTRA and its derivatives with Mg^2+^ and Ca^2+^ [9]. The dissociation constants *K*_d_ values so obtained are summarized in Table 4. In each case, the values for Mg^2+^ were in the mM range and for Ca^2+^ in the µM range, which is consistent with other reported APTRA derivatives. However, three clear trends emerge according to the isomeric form (*p* or *m*) and the identity of the substituent:

1. The *m* compounds are seen to have higher metal binding affinities—for both Mg^2+^ and Ca^2+^—than the corresponding *p* isomers.
2. Within each series (*p* or *m*), the M^2+^ affinities—again for both Mg^2+^ and Ca^2+^—decrease as the substituent becomes more electron‐withdrawing, that is, *K*_d_ values increase from OMe to CF_3_ to CN.
3. The second effect is proportionately greater for Ca^2+^ than for Mg^2+^, with the result that the *selectivity* toward Mg^2+^ is improved. Table 4 uses *K*_a_(Mg^2+^) / *K*_a_(Ca^2+^) as a quantifier of selectivity toward Mg^2+^ (where *K*_a_ is the 1:1, M:L association constant = 1/*K*_d_), from which it is seen that the selectivity almost doubles on going from OMe to CN.

**TABLE 4:** *K*_d_ values for the *p* and *m* series of ligands binding to Mg^2+^ and Ca^2+^ in buffered aqueous solution (pH 7.2) in the presence of 0.1 M KCl at 295 K, evaluated by absorption spectroscopy.^[^ ^a^ ^]^

Ligand	*K*_d_ Mg^2+^ / mM	*K*_d_ Ca^2+^ / µM	Mg^2+^ selectivity^[^ ^b^ ^]^ / 10^−3^
CN‐*p*	9.1 ± 0.4	74 ± 1	8.1
CF_3_‐*p*	8.1 ± 0.6	62 ± 4	7.7
OMe‐*p*	7.1 ± 0.2	33 ± 2	4.7
CN‐*m*	5.2 ± 0.3	39 ± 1	7.5
CF_3_‐*m*	3.7 ± 0.6	20 ± 1	5.5
OMe‐*m*	3.2 ± 0.5	13 ± 3	4.1

^a^The standard error on the mean *K*_d_ from at least three separate titrations is indicated.

^b^Mg^2+^ selectivity quantified as the relative association constants *K*_a_ for Mg^2+^ and Ca^2+^, where *K*_a_ = *K*_d_^−1^. The corresponding value for APTRA itself is 5.4 × 10^−3^, using the data from Table 1.

### 2.6 Interpretation of the Trends in *K*_d_

APTRA is a pentadentate ligand that probably binds to the target metal ion M^2+^ in solution through the anilino nitrogen atom, the phenolic oxygen atom, and the three carboxylates. This pentadentate binding mode is supported by X‐ray diffraction studies in the solid state by Buccella and coworkers, with the sixth coordination site occupied by a water molecule [9]. The decline in metal ion affinity with substituent in the two series in the order OMe > CF_3_> CN is, then, to be expected, since electron‐withdrawing groups will deplete the ground‐state electron density at the nitrogen and phenolic oxygen atoms of the APTRA unit, suppressing their ability to bind to the cationic metal ion. However, the higher electronegativity of oxygen versus nitrogen, and its poorer orbital overlap with the aromatic π system, (Hammett *σ*_p_ values for OMe and NMe_2_ {models for –OCH_2_ and –N(CH_2_)_2_ in APTRA} are –0.27 and –0.83, respectively) will mean that compounds in which the electron‐withdrawing ─C≡C─C_6_H_4_–R group is positioned *para* to oxygen will be affected less than the isomers where the group is *para* to nitrogen. Thus, the affinities are lowered less (relative to APTRA) in the *m* series than in the *p* series. Perhaps the most interesting trend, though, is the increase in *selectivity* for Mg^2+^ with electron‐withdrawing power of the substituent. This probably reflects the relative importance of the nitrogen atom in the binding of the M^2+^ ion. The smaller, more charge‐dense, and thus “harder” Mg^2+^ ion may rely more on the harder oxygen donors (the phenolic O, the carboxylates, and bound water) than on ligation by the nitrogen atom, whereas Ca^2+^ is expected to have a greater interaction with the anilino nitrogen lone pair. Thus, Ca^2+^ binding will be suppressed to a greater extent than Mg^2+^ by electron‐withdrawing substituents, leading to the observed effect on selectivity.

### 2.7 Discussion and Concluding Remarks

The work described here cements a straightforward synthetic approach to the appendage of aryl azacarboxylate ligands like APTRA with aryl‐alkynyl fluorophores. The resulting structures may be regarded as derivatives of DPA. We have highlighted the difference in excited‐state properties of the resulting DPA unit according to the identity of the substituent on the aryl‐alkynyl unit. Thus, whilst electron‐withdrawing substituents like CN and CF_3_ lead to highly emissive ICT states, the emission is much weaker in methoxy‐substituted analogues. Nevertheless, despite the respectable fluorescence quantum yields associated with the CN and CF_3_ esters, the corresponding carboxylates emit only weakly in water, and the displacement of charge away from the APTRA ring that accompanies the formation of the ICT state leads to rather minimal changes in fluorescence upon metal binding.

On the other hand, the ground‐state absorption properties prove more useful, and they provide deeper insight into the influence of substituents on the selectivity for Mg^2+^ versus Ca^2+^. The key conclusion is that the more strongly electron‐withdrawing the substituent in the arylalkynyl unit, the weaker the binding of metal ions at the APTRA ring. But, the affinity for Ca^2+^ is attenuated more than Mg^2+^, such that the selectivity toward Mg^2+^ is enhanced. The effect is observed irrespective of whether the alkyne is positioned *para* or *meta* to the APTRA amino group. The *meta* compounds display stronger binding than their *para* isomers in all cases, in line with the electron density at nitrogen being depleted to a lesser extent. However, by the same token, superior Mg^2+^ selectivities are achieved using the *para* isomers. They also show a much greater perturbation in the absorption spectra, owing to the greater D–A character.

Whilst we conclude from our study that arylalkynyl derivatives of ligands like APTRA are probably largely unsuitable for further study in fluorescent sensing owing to the limitations discussed, it does not rule out their utility using other sensing methods. For example, the substantial effect seen on the absorptive transition for the *p* isomers could translate into perturbations in NLO properties [54], while the C≡C stretching band probed through IR or Raman spectroscopy may prove to be a rich resource [55, 56].

More importantly, we conclude that electronic effects of substituents in aryl‐azacarboxylate ligands on the relative binding affinities for metal ions may be quite general. As noted in the introduction, the ability to selectively perturb metal ion concentrations—especially the relative values of Mg^2+^ and Ca^2+^—using tailor‐made, cell‐permeable ligands opens up new prospects in contemporary research areas such as chromatin structure and gene expression. In the present instance, the overall binding strength is effectively controlled through the choice of regioisomer, and the selectivity can then be fine‐tuned further according to the identity of the substituent. Access to cell‐permeable derivatives can be readily envisaged through the formation of acetoxymethyl (AM) esters [37], to allow direct application in future live‐cell studies.

## 3 Experimental Details

### 3.1 General Synthetic Procedures

*p*‐Br‐APTRA‐Et_3_ was prepared and converted to *p*‐TiPSA‐APTRA‐Et_3_ as described previously [19]. *m*‑TiPSA‐APTRA‐Et_3_ was likewise prepared as we reported previously, but the precursor *m*‐Br‐APTRA‐Et_3_ was obtained using a different procedure from that used before (see Supporting Information). The synthetic procedures and characterization for the esters are given below. Data for the carboxylates are given in the Supporting Information. All commercially available reagents were used as supplied, without further purification. Air‐ and moisture‐sensitive reactions were carried out under a nitrogen atmosphere using Schlenk‐line techniques. Thin‐layer chromatography was performed on silica (Merck Art 5554) and visualized under UV irradiation at 254 nm. Column chromatography was performed on combi‐flash instruments using RediSep R_f_ silica cartridges. ^1^H, ^13^C{^1^H} and ^19^F{^1^H} NMR spectra of the final compounds and of their immediate ester precursors, including 2‐dimensional spectra, were recorded on either a Varian VNMRS 600 MHz or a Bruker Neo 700 MHz spectrometer. Electrospray mass spectra were recorded on a Waters SQD instrument, interfaced with an Acquity UPLC system.

### 3.2 General Procedure for the Sonogashira Coupling of *p*‐ or *M*‐TiPSA‐APTRA‐Et_3_ With *Para*‐Substituted Aryl Iodides

Anhydrous tetrahydrofuran (3 mL per mmol of acetylide) was added to *p*‐ or *m*‐TiPSA‐APTRA‐Et_3_ and the *para*‐substituted aryl iodide (1.1 equiv.) in a Schlenk tube. The solution was degassed by three freeze‐pump‐thaw cycles and then filled with nitrogen gas. Pd(dppf)Cl_2_ (0.15 equiv.), CuI (0.30 equiv.) and NEt_3_ (8.0 equiv.) were added under a flow of nitrogen. The mixture was degassed by a further three freeze‐pump‐thaw cycles and filled with nitrogen. Finally, *n*‐Bu_4_NF (1.4 equiv. in the form of a 1 M solution in THF) was added, the degassing procedure was repeated, and the reaction was refluxed under nitrogen for 24 h. The solvent was removed under reduced pressure, and the product was obtained from the residue by column chromatography on silica, using the conditions specified for each compound below.

### 3.3 CN‐*p*‐Et_3_ Diethyl 2,2′‐((4‐((4‐cyanophenyl)ethynyl)‐2‐(2‐ethoxy‐2‐oxoethoxy)phenyl)Azanediyl) Diacetate

This compound was prepared from *p*‐TiPSA‐APTRA‐Et_3_ (0.23 g, 0.42 mmol) and 4­iodobenzonitrile (0.11 g, 0.46 mmol) using the general procedure described above. Chromatography involved a gradient elution using hexanes / ethyl acetate from 100:0 to 70:30, giving the product as a viscous brown oil (140 mg, 67 %); R_f_ (80:20)  =  0.1. ^1^H NMR (600 MHz, CDCl_3_) *δ*  =  7.61 – 7.59 (2H, m, H^19^), 7.56 – 7.53 (2H, m, H^18^), 7.10 (1H, dd, J 8.3, 1.8, H^7^), 6.93 (1H, d, J 1.8, H^9^), 6.79 (1H, d, J 8.3, H^6^), 4.62 (2H, s, H^11^), 4.25 (2H, q, J 7.2, H^13^), 4.22 – 4.16 (8H, m, H^2^ and H^4^), 1.30 – 1.23 (9H, m, H^1^ and H^14^). ^13^C (151 MHz, CDCl_3_) *δ*  =  171.0 (C^3^), 168.5 (C^12^), 148.8 (C^10^), 140.8 (C^8^), 132.0 (C^19^), 131.8 (C^18^), 128.4 (C^20^ or C^21^), 126.7 (C^7^), 118.9 (C^6^), 118.6 (C^20^ or C^21^), 117.5 (C^9^), 115.0 (C^5^), 111.1 (C^17^), 94.0 (C^15^), 87.0 (C^16^), 66.2 (C^11^), 61.3 (C^13^), 60.9 (C^2^), 53.8 (C^4^), 14.2 (C^1^), 14.1 (C^14^). ESI *m/z* = 493.42 ([C_27_H_28_N_2_O_7_ + H]^+^, 100 %); HRMS calculated for [C_27_H_29_N_2_O_7_]^+^ 493.1982, found 493.1975.


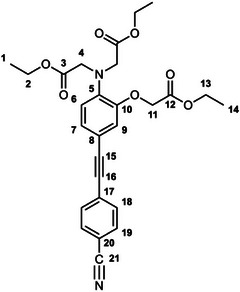


### 3.4 CF_3_‐*p*‐Et_3_ Diethyl 2,2′‐((2‐(2‐ethoxy‐2‐oxoethoxy)‐4‐((4‐(trifluoromethyl)phenyl)Ethynyl)Phenyl) Azanediyl)Diacetate

This compound was prepared from *p*‐TiPSA‐APTRA‐Et_3_ (0.22 g, 0.41 mmol) and 4­iodobenzotrifluoride (0.07 mL, 0.5 mmol) using the general procedure described above. Chromatography involved a gradient elution using hexanes / ethyl acetate from 100:0 to 80:20, giving the product as a viscous brown oil (110 mg, 49 %); R_f_ (80:20)  =  0.3. ^1^H (600 MHz, CDCl_3_) *δ*  =  7.58 – 7.56 (4H, m, H^18^ and H^19^), 7.11 (1H, dd, J 8.3, 1.8, H^7^), 6.94 (1H, d, J 1.8, H^9^), 6.79 (1H, d, J 8.3, H^6^), 4.63 (2H, s, H^11^), 4.25 (2H, q, J 7.2, H^13^), 4.22 – 4.16 (8H, m, H^2^ and H^4^), 1.29 (3H, t, J 7.2, H^14^), 1.25 (6H, t, J 7.2, H^1^). ^19^F (376 MHz, CDCl_3_) *δ*  = − 62.7 (CF_3_). ^13^C (151 MHz, CDCl_3_) *δ*  =  171.1 (C^3^), 168.5 (C^12^), 148.8 (C^10^), 140.5 (C^5^), 131.6 (C^18^ or C^19^), 129.4 (C^17^, C^20^ or C^21^), 127.3 (C^17^, C^20^ or C^21^), 126.5 (C^7^), 125.2 (C^18^ or C^19^), 123.0 (C^17^, C^20^ or C^21^), 119.0 (C^6^), 117.5 (C^9^), 115.4 (C^8^), 91.8 (C^15^), 87.1 (C^16^), 61.3 (C^13^), 61.2 (C^11^), 60.8 (C^2^), 53.7 (C^4^), 14.2 (C^1^), 14.1 (C^14^). ESI *m/z*  =  536.36 ([C_27_H_28_F_3_NO_7_ + H]^+^, 100 %); HRMS calculated for [C_27_H_29_F_3_NO_7_]^+^ 536.1916, found 536.1896.


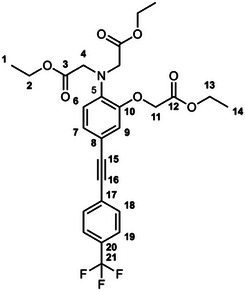


### 3.5 OMe‐*p*‐Et_3_ Diethyl 2,2′‐((2‐(2‐ethoxy‐2‐oxoethoxy)‐4‐((4‐methoxyphenyl)ethynyl)Phenyl) Azanediyl)Diacetate

This compound was prepared from *p*‐TiPSA‐APTRA‐Et_3_ (0.15 g, 0.27 mmol) and 4­iodoanisole (70 mg, 0.30 mmol) using the general procedure described above. Chromatography involved a gradient elution using hexanes / ethyl acetate from 100:0 to 70:30, giving the product as a greenish oil (20 mg, 13 %); R_f_ (80:20)  =  0.2. ^1^H (600 MHz, CDCl_3_) *δ*  =  7.43 – 7.40 (2H, m, H^18^), 7.07 (1H, dd, J 8.3, 1.8, H^7^), 6.91 (1H, d, J 1.8, H^9^), 6.86 – 6.83 (2H, m, H^19^), 6.79 (1H, d, J 8.3, H^6^), 4.63 (2H, s, H^11^), 4.24 (2H, q, J 7.2, H^13^), 4.21 – 4.15 (8H, m, H^2^ and H^4^), 1.28 (3H, t, J 7.1, H^14^), 1.24 (6H, t, J 7.2, H^1^). ^13^C (151 MHz, CDCl_3_) *δ*  =  171.1 (C^3^), 168.6 (C^12^), 159.4 (C^20^), 148.9 (C^10^), 139.7 (C^5^), 132.8 (C^18^), 126.1 (C^7^), 119.1 (C^9^), 117.2 (C^6^), 116.8 (C^8^), 115.5 (C^17^), 113.9 (C^19^), 88.4 (C^16^), 87.9 (C^15^), 66.1 (C^11^), 61.2 (C^13^), 60.8 (C^2^), 55.3 (C^21^), 53.8 (C^4^), 14.2 (C^1^), 14.1 (C^14^). ESI *m/z* = 498.35 ([C_27_H_31_NO_8_ + H]^+^, 100 %); HRMS calculated for [C_27_H_32_NO_8_]^+^ 498.2112, found 498.2128.


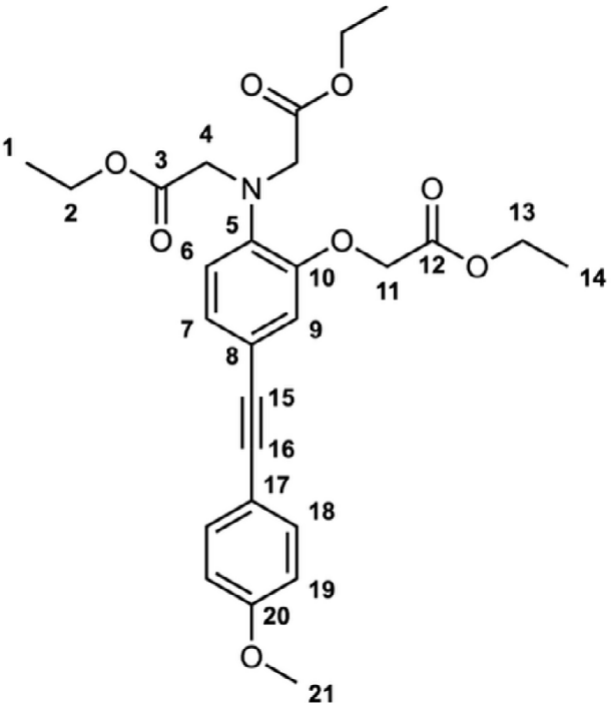


### 3.6 CN‐*m*‐Et_3_ Diethyl 2,2′‐((5‐((4‐cyanophenyl)ethynyl)‐2‐(2‐ethoxy‐2‐oxoethoxy) phenyl)Azanediyl)Diacetate

This compound was prepared from *m*‐TiPSA‐APTRA‐Et_3_ (53 mg, 0.097 mmol) and 4­iodobenzonitrile (25 mg, 0.11 mmol) using the general procedure described above. Chromatography involved a gradient elution using hexanes / ethyl acetate from 100:0 to 70:30, giving the product as an off‐white solid (22 mg, 46 %); R_f_ (80:20)  =  0.1. ^1^H (600 MHz, CDCl_3_) *δ*  =  7.61 – 7.59 (2H, m, H^19^), 7.55 – 7.53 (2H, m, H^18^), 7.10 – 7.06 (2H, m, H^7^ and H^9^), 6.73 (1H, d, J 8.2, H^6^), 4.66 (2H, s, H^4^), 4.23 (2H, q, J 7.2, H^2^), 4.20 – 4.16 (8H, m, H^11^ and H^13^), 1.29 – 1.22 (9H, m, H^1^ and H^14^). ^13^C (151 MHz, CDCl_3_) *δ*  =  171.0 (C^12^), 168.4 (C^3^), 150.6 (C^5^), 139.5 (C^10^), 132.0 (C^19^), 131.9 (C^18^), 128.4 (C^20^ or C^21^), 126.4 (C^6^ or C^7^), 123.4 (C^6^ or C^7^), 118.6 (C^20^ or C^21^), 116.0 (C^8^), 114.0 (C^9^), 111.1 (C^17^), 93.9 (C^15^), 86.6 (C^16^), 65.9 (C^4^), 61.2 (C^2^), 60.8 (C^13^), 53.6 (C^11^), 14.2 (C^14^), 14.10 (C^1^). ESI *m/z* = 493.42 ([C_27_H_28_N_2_O_7_ + H]^+^, 100 %); HRMS calculated for [C_27_H_29_N_2_O_7_]^+^ 493.1953, found 493.1975.


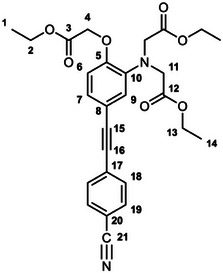


### 3.7 CF_3_‐*m*‐Et_3_ Diethyl 2,2′‐((2‐(2‐ethoxy‐2‐oxoethoxy)‐5‐((4‐(trifluoromethyl)phenyl)Ethynyl)Phenyl) Azanediyl)Diacetate

This compound was prepared from *m*‐TiPSA‐APTRA‐Et_3_ (0.15 g, 0.27 mmol) and 4­iodobenzo‐trifluoride (0.04 mL, 0.3 mmol) using the general procedure described above. Chromatography involved a gradient elution using hexanes / ethyl acetate from 100:0 to 80:20, giving the product as an orange oil (110 mg, 74 %); R_f_ (80:20)  =  0.2. ^1^H (600 MHz, CDCl_3_) *δ*  =  7.60 – 7.58 (4H, m, H^18^ and H^19^), 7.10 – 7.08 (2H, m, H^7^ and H^9^), 6.74 (1H, d, J 8.0, H^6^), 4.66 (2H, s, H^4^), 4.24 (2H, q, J 7.2, H^2^), 4.21 – 4.16 (8H, m, H^11^ and H^13^), 1.28 – 1.24 (9H, m, H^1^ and H^14^). ^19^F (376 MHz, CDCl_3_) *δ*  =  − 62.7 (CF_3_). ^13^C (151 MHz, CDCl_3_) *δ*  =  171.0 (C^12^), 168.4 (C^3^), 150.4 (C^5^), 139.5 (C^10^),131.6 (C^18^ or C^19^), 129.6 (C^17^, C^20^ or C^21^), 127.3 (C^17^, C^20^ or C^21^), 126.3 (C^7^ or C^9^), 125.2 (C^18^ or C^19^), 123.4 (C^7^ or C^9^), 123.1 (C^17^, C^20^ or C^21^), 114.0 (C^6^), 91.8 (C^15^), 86.9 (C^16^), 66.0 (C^4^), 61.3 (C^2^), 60.7 (C^13^), 53.7 (C^11^), 14.2 (C^14^), 14.1 (C^1^). The signal for C^8^ is too weak to identify. ESI *m/z* = 536.36 ([C_27_H_28_F_3_NO_7_ + H]^+^, 100 %); HRMS calculated for [C_27_H_29_F_3_NO_7_]^+^ 536.1896, found 536.1896.


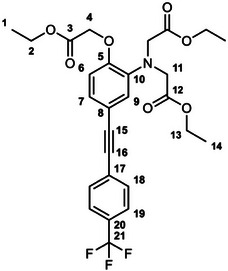


### 3.8 OMe‐*m*‐Et_3_ Diethyl 2,2′‐((2‐(2‐ethoxy‐2‐oxoethoxy)‐5‐((4‐methoxyphenyl)ethynyl)Phenyl)Azanediyl) Diacetate

This compound was prepared from *m*‐TiPSA‐APTRA‐Et_3_ (77 mg, 0.14 mmol) and 4­iodoanisole (37 mg, 0.16 mmol) using the general procedure described above. Chromatography involved a gradient elution using hexanes / ethyl acetate from 100:0 to 75:25, giving the product as a green‐brown oil (29 mg, 42 %); R_f_ (80:20)  =  0.2. ^1^H NMR (600 MHz, CDCl_3_) *δ*  =  7.47 – 7.44 (2H, m, H^18^), 7.10 – 7.08 (2H, m, H^7^ and H^9^), 6.90 – 6.87 (2H, m, H^19^), 6.72 (1H, d, J 8.9, H^6^), 4.69 (2H, s, H^4^), 4.30 – 4.16 (10H, m, H^2^, H^11^ and H^13^), 3.81 (3H, s, H^21^), 1.33 – 1.26 (9H, m, H^1^ and H^14^). ^13^C (151 MHz, CDCl_3_) *δ*  =  171.1 (C^12^), 168.6 (C^3^), 159.4 (C^20^), 149.9 (C^5^), 139.4 (C^10^), 132.9 (C^18^), 125.9 (C^7^ or C^9^), 123.2 (C^7^ or C^9^), 117.6 (C^8^), 115.5 (C^17^), 114.1 (C^6^), 113.9 (C^19^), 88.1 (C^16^), 87.9 (C^15^), 66.1 (C^4^), 61.2 (C^2^), 60.7 (C^13^), 55.3 (C^21^), 53.6 (C^11^), 14.2 (C^14^), 14.1 (C^1^). ESI *m/z* = 498.44 ([C_27_H_31_NO_8_ + H]^+^, 100 %); HRMS calculated for [C_27_H_32_NO_8_]^+^ 498.2114, found 498.2128.


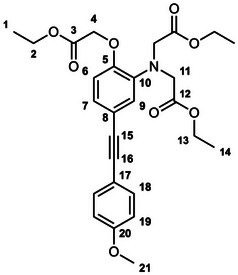


### 3.9 Ester Hydrolysis to Form the Carboxylates

The procedure for obtaining samples for characterization is described in the Supporting Information, together with full NMR and mass spectrometric data for each of the six ligands. Samples of the ligands for the determination of *K*_d_ values with Mg^2+^ and Ca^2+^ were obtained as and when required by adding the ester to a mixture of aqueous NaOH (1 M, 6.25 mL per mmol of ester) and methanol (25 mL per mmol of ester). The solution was stirred at room temperature for 1 h and then diluted to 5 mL with aqueous buffer (50 mM HEPES, pH 7.2, containing 0.1 M KCl). Complete hydrolysis was confirmed by ESI‐LRMS. Appropriate aliquots of this 5 mL stock solution were then used to form solutions of known ligand concentration.

### 3.10 Optical Spectroscopy

Absorption spectra were recorded with a Uvikon XS double‐beam spectrometer operated through LabPower Software. Samples were contained in quartz cuvettes with a pathlength of 1 cm relative to the same solvent as reference; spectra were collected at 1 nm increments at a rate of 200 nm / min. Fluorescence spectra were acquired using either a Jobin Yvon Fluoromax‐2 or FluoroLog‐3 spectrometer, with a Hamamatsu R928 detector in both cases. Samples were contained in quartz cuvettes of 1 cm pathlength. Spectra were collected at 1 nm increments and integration time of 0.5 s. Quantum yields were determined using quinine sulfate in aqueous H_2_SO_4_ (1 M) as the standard, for which Φ_f_ = 0.546 [57]. Fluorescence lifetimes were determined by time‐correlated single‐photon counting (TCSPC) using an Edinburgh Instruments OB920 spectrometer following excitation at 374 nm with a pulsed laser diode.

### 3.11 Spectrophotometric Titrations and Determination of *K*_d_

All metal ion binding studies were carried out in buffered aqueous (50 mM HEPES, pH 7.2) in the presence of KCl (0.1 M) to ensure that the ionic strength remains roughly constant. To a solution of the ligand in the absorption cuvette (25 µM, 2 mL), appropriate aliquots of the metal solution {[Mg^2+^] =  200 mM, [Ca^2+^] =  2 mM} were gradually added. The metal solutions also contained the same concentration of the ligand to avoid issues with ligand dilution over the course of the titration. After the addition of each aliquot, the solution was mixed by inversion of the cuvette and allowed to equilibrate for 5 min before acquisition of the absorption spectrum. Aliquots were added until 1 mL total of the metal solution had been added to achieve metal saturation conditions: [Mg^2+^] =  67 mM, [Ca^2+^] =  667 µM. A wavelength showing a suitable gradual change over the course of the titration was selected for monitoring; the absorbance at this wavelength was plotted against [M^2+^] and fitted using a 1:1 binding model [58, 59]. Quoted *K*_d_ values are the mean of at least three fully independent titrations, with the standard error indicated.

## Conflicts of Interest

The authors declare no competing financial interest.

## Supporting information



Electronic supporting information is available: further synthetic details and characterization data for the ligands; ^1^H and ^13^C NMR spectra; additional absorption and emission spectra. The Supporting Information cites only reference 19 from the main text; there are no additional references.

## Data Availability

The data that support the findings of this study are available in the supplementary material of this article.
