# Supplementary material for: Fluorescence Properties and Mg2+ Selectivity of Aryl‐Alkynyl Derivatives of the o‐Aminophenoltriacetate (APTRA) Ligand
Source: Chemistry. 2026 Jan 7;32(10):e03428. doi: 10.1002/chem.202503428 (PMC12995852; doi:10.1002/chem.202503428)
Supplement: Supplementary file 1 — Electronic supporting information is available: further synthetic details and characterization data for the ligands; 1H and 13C NMR spectra; additional absorption and emission spectra. The Supporting Information cites only reference 19 from the main text; there are no additional references. [file CHEM-32-e03428-s001.pdf]

Fluorescence Properties and Mg^{2+} Selectivity of Aryl-alkynyl Derivatives of the *o*-Aminophenoltriacetate (APTRA) Ligand

Laura L. Duncan, Christopher Hogg and J. A. Gareth Williams*

Department of Chemistry, Durham University, Durham, DH1 3LE, U.K.
** E-mail: j.a.g.williams@durham.ac.uk*

Contents

Section 1. Additional synthetic details: route to the *p* family and synthesis of precursors.... **Page S2**

Section 2. Characterization data for the ligands – as their carboxylic acids..................... **Page S3**

Section 3. Metal binding studies with Mg^{2+} and Ca^{2+}.. **Page S6**

Section 4. NMR spectra of the ester precursors and the final carboxylate ligands.............. **Page S11**

1. Additional synthetic details: route to the *p* family and synthesis of precursors

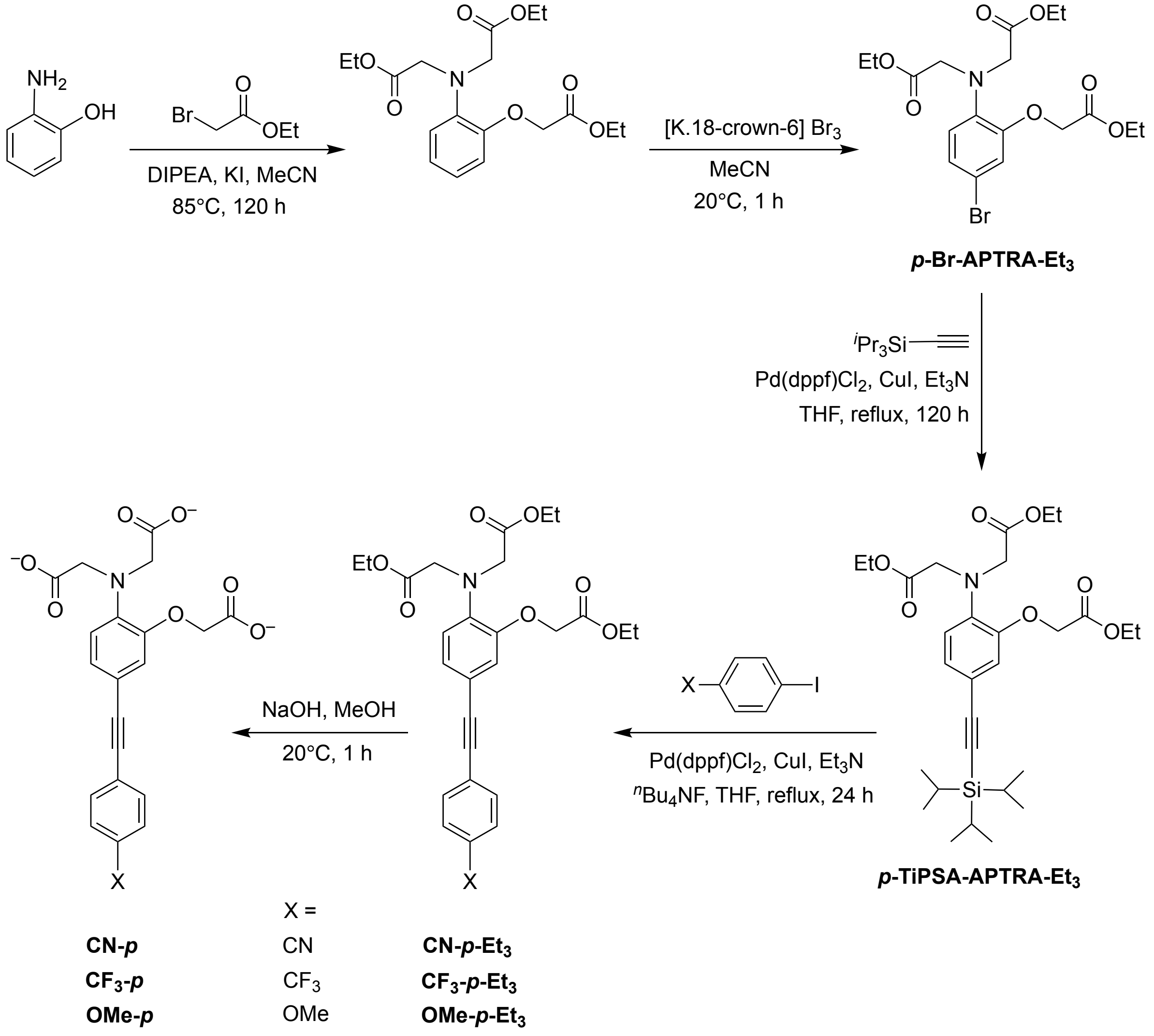

Scheme S1. *Synthetic route to the para-linked family of compounds CF3-p, CN-p, OMe-p. The first two steps, to p-Br-APTRA-Et, followed the method described previously.*[19]

Synthesis of *m*-Br-APTRA-Et$_3$

Diethyl 2,2'-((5-bromo-2-(2-ethoxy-2-oxoethoxy)phenyl)azanediyl) diacetate

A solution of 2-amino-4-bromophenol (1.8 g, 9.8 mmol), chloroacetic acid (4.7 g, 50 mmol) and NaOH (2.8 g, 69 mmol) in water (10 mL) was refluxed for 3 h. One further pellet of NaOH was added every 15 min. The water was then removed under reduced pressure to give a light brown oil, containing crude *m*-Br-APTRA, which was esterified directly. Thus, the oil was taken up into ethanol (35 mL) and concentrated sulphuric acid (7.6 mL) was added, and the solution was refluxed for 7 h. The ethanol was then removed under reduced pressure, and the crude product

19. E. R. H. Walter, J. A. G. Williams and D. Parker, Tuning Mg(II) selectivity: Comparative analysis of the photophysical properties of four fluorescent probes with an alkynyl-naphthalene fluorophore. *Chem. Eur. J.*, 2018, **24**, 6432–6441.

was dissolved in ethyl acetate and washed with aqueous NaOH (2.5 M) and brine before drying over anhydrous $MgSO_4$. The solvent was removed under reduced pressure, and the residue was subjected to column chromatography on silica (gradient elution using hexanes / ethyl acetate from 100:0 to 85:15), giving the product as a brown solid (1.2 g, 29 %).

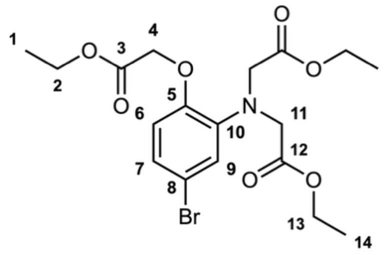

R_f (80 % hexanes, 20 % ethyl acetate) = 0.2. ^{1}H NMR (400 MHz, $CDCl_3$) δ = 7.03–6.70 (2H, m, H^7 and H^9), 6.71–6.67 (1H, m, H^6), 4.63 (2H, s, H^4), 4.29–4.18 (10H, m, H^2, H^{11} and H^{13}), 1.33–1.26 (9H, m, H^1 and H^{14}). ESI-LRMS *m/z* = 446.37 ([$C_{18}H_{24}{}^{79}BrNO_7$ + H]$^+$, 99 %), 448.39 ([$C_{18}H_{24}{}^{81}BrNO_7$ + H]$^+$, 100). Spectra in agreement with literature data.[1]

Synthesis of *m*-TiPSA-APTRA-Et$_3$ and *p*-TiPSA-APTRA-Et$_3$

These two intermediates were prepared from *m*- and *p*-Br-APTRA-Et$_3$, respectively, as described previously.[op. cit.]

2. Characterization data for the ligands – as their carboxylic acids – prepared by hydrolysis of the ethyl esters

The ester was added to a mixture of aqueous NaOH (1 M, 10 mL per mmol of ester) and methanol (20 mL) and the resulting solution was stirred at room temperature for 24 h. More water was added, and the solution was neutralised with HCl (1 M) before removal of the solvent under reduced pressure. Complete hydrolysis was confirmed by ESI-LRMS. NMR and mass spectrometric characterisation data for the individual compounds are compiled below.

CN-*p*

2,2'-((2-(carboxymethoxy)-4-((4-cyanophenyl)ethynyl)phenyl)azanediyl)diacetic acid

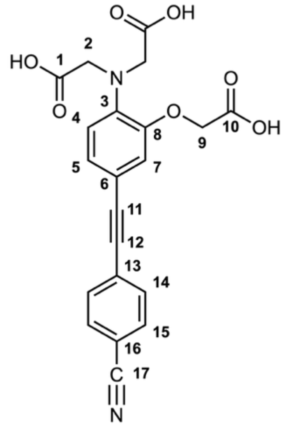

^{1}H (700 MHz, D_2O) δ = 7.70 – 7.68 (2H, m, H^{15}), 7.60 – 7.57 (2H, m, H^{14}), 7.09 (1H, d, J 8.3, H^5), 6.90 (1H, s, H^7), 6.75 – 6.67 (1H, m, H^4), 4.41 (2H, s, H^9), 3.83 (4H, s, H^2). ^{13}C (176 MHz, D_2O) δ = 179.5 (C^{10}), 177.2 (C^1), 148.7 (C^3), 141.4 (C^8), 132.5 (C^{15}), 131.7 (C^{14}), 128.3 (C^{16} or C^{17}), 125.9 (C^5), 119.6 (C^{16} or C^{17}), 117.2 (C^4), 116.1 (C^7), 110.0 (C^{13}), 87.1 (C^{12}), 67.8 (C^2), 56.8 (C^9). Signals for C^6 and C^{11} were too weak to assign. ESI-LRMS *m/z* = 409.36 ([$C_{21}H_{16}N_2O_7$ + H]$^+$, 100 %). ESI-HRMS calculated for [$C_{21}H_{17}N_2O_7$]$^+$ 409.1031, found 409.1036.

CF$_3$-*p*

2,2'-((2-(carboxymethoxy)-4-((4-(trifluoromethyl)phenyl)ethynyl)phenyl)azanediyl) diacetic acid

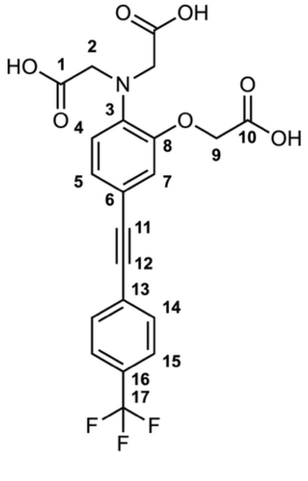

^{1}H (600 MHz, D_2O) δ = 7.66 – 7.53 (4H, m, H^{14} and H^{15}), 7.08 (1H, d, J 8.2, H^5), 6.91 (1H, s, H^7), 6.74 (1H, d, J 8.3, H^4), 4.40 (2H, s, H^9), 3.76 (4H, s, H^2). ^{13}C (151 MHz, D_2O) δ = 179.3 (C^1), 177.0 (C^{10}), 149.0 (C^8), 141.2 (C^3), 131.6 (C^{14} or C^{15}), 125.7 (C^5), 125.5 (C^{14} or C^{15}), 117.6 (C^4), 116.0 (C^7), 92.0 (C^{11} or C^{12}), 87.1 (C^{11} or C^{12}), 67.6 (C^9) and 57.0 (C^2). Signals for C^6, C^{13}, C^{16}, C^{17} were too weak to assign. ^{19}F (376 MHz, D_2O) δ = − 62.46. ESI-LRMS *m/z* = 452.26 ([$C_{21}H_{16}F_3NO_7$ + H]$^+$, 100 %). ESI-HRMS calculated for [$C_{21}H_{17}F_3NO_7$]$^+$ 452.0957 found 452.0957.

OMe-*p*

2,2'-((2-carboxymethoxy)-4-((4-methoxyphenyl)ethynyl)phenyl)azanediyl)diacetic acid

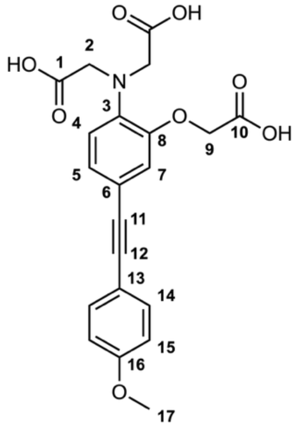

^{1}H (600 MHz, D_2O) δ = 7.41 – 7.37 (2H, m, H^{14}), 6.99 (1H, dd, J 8.3, 1.6, H^5), 6.88 (2H, d, J 8.8, H^{15}), 6.85 – 6.83 (1H, m, H^7), 6.75 (1H, d, J 8.3, H^4), 4.37 (2H, s, H^{16}), 3.73 (3H, s, H^{17}), 3.71 (4H, s, H^2). ^{13}C (151 MHz, D_2O) δ = 179.3 (C^1), 176.8 (C^{10}), 159.0 (C^{16}), 149.2 (C^8), 140.5 (C^3), 132.9 (C^{14}), 125.1 (C^5), 118.0 (C^4), 115.6 (C^7), 115.0 (C^6 or C^{13}), 114.4 (C^{15}), 88.5 (C^{11}), 88.2 (C^{12}), 67.4 (C^9), 57.1 (C^2), 55.2 (C^{17}). ESI-LRMS *m/z* 414.33 ([$C_{21}H_{19}NO_8$ + H]$^+$, 100 %). ESI-HRMS calculated for [$C_{21}H_{20}NO_8$]$^+$ 414.1182 found 414.1189.

CN-*m*

2,2'-((2-(carboxymethoxy)-5-((4-cyanophenyl)ethynyl)phenyl)azanediyl)diacetic acid

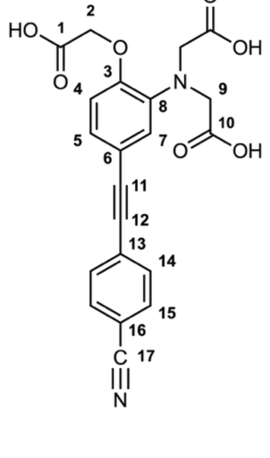

^{1}H (600 MHz, D_2O) δ = 7.65 – 7.62 (2H, m, H^{15}), 7.55 – 7.52 (2H, m, H^{14}), 7.04 – 7.00 (1H, m, H^5), 6.95 (1H, s, H^7), 6.67 (1H, d, J 8.5, H^4), 4.38 (2H, s, H^2), 3.70 (4H, s, H^9). ^{13}C (150 MHz, D_2O) δ = 179.4 (C^{10}), 176.8 (C^1), 151.0 (C^3), 139.9 (C^8), 132.4 (C^{15}), 131.7 (C^{14}), 128.1 (C^{16} or C^{17}), 125.6 (C^5), 121.3 (C^7), 119.5 (C^{16} or C^{17}), 112.9 (C^4), 88.7 (C^{11}), 86.6 (C^{12}), 67.3 (C^2), 56.8 (C^9). ESI-LRMS *m/z* 409.24 ([$C_{21}H_{16}N_2O_7$ + H]$^+$, 100 %). ESI-HRMS calculated for [$C_{21}H_{17}N_2O_7$]$^+$ 409.1033 found 409.1036.

CF_3-*m*

2,2'-((2-(carboxyoxy)-5-((4-(trifluoromethyl)phenyl)ethynyl)phenyl)azaneidiyl)diacetic acid

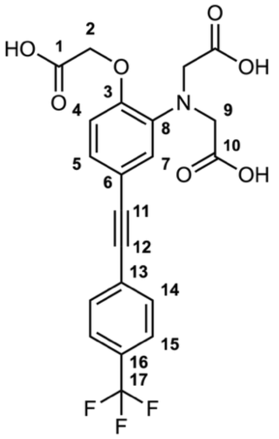

^{1}H (600 MHz, D_2O) δ = 7.61 – 7.53 (4H, m, H^{14} and H^{15}), 7.02 (1H, d, J 8.5, H^5), 6.96 (1H, s, H^7), 6.67 (1H, d, J 8.5, H^4), 4.34 (2H, s, H^2), 3.68 (4H, s, H^9). ^{13}C (151 MHz, D_2O) δ = 179.3 (C^{10}), 176.8 (C^1), 150.8 (C^3), 139.9 (C^8), 131.6 (C^{14} or C^{15}), 125.8 (C^5), 125.4 (C^{14} or C^{15}), 121.3 (C^7), 112.9 (C^4), 91.9 (C^{11}), 86.8 (C^{12}), 67.2 (C^2), 56.8 (C^9). Signals for C^6, C^{13}, C^{16}, C^{17} were too weak to assign. ^{19}F (376 MHz, D_2O) δ = – 62.5. ESI-LRMS *m/z* 452.22 ($[C_{21}H_{16}F_3NO_7 + H]^+$, 53 %). ESI-HRMS calculated for $[C_{21}H_{17}F_3NO_7]^+$ 452.0950 found 452.0957.

OMe-*m*

2,2'-((2-(carboxymethoxy)-5-((4-methoxyphenyl)ethynyl)phenyl)azanediyl)diacetic acid

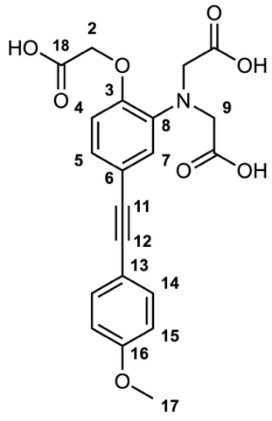

^{1}H (700 MHz, D_2O) δ = 7.46 (2H, d, J 8.7, H^{14}), 7.03 (1H, d, J 8.0, H^5), 6.98 – 6.93 (3H, m, H^7 and H^{15}), 6.71 (1H, d, J 8.3, H^4), 4.43 (2H, s, H^2), 3.79 (3H, s, H^{17}), 3.76 (4H, s, H^9). ^{13}C (176 MHz, D_2O) δ = 179.5 (C^{10}), 177.0 (C^1), 159.0 (C^{16}), 150.3 (C^3), 139.9 (C^8), 132.9 (C^{14}), 125.0 (C^5), 120.9 (C^7), 115.1 (C^6), 114.5 (C^{15}), 113.1 (C^4), 88.6 (C^{11}), 87.8 (C^{12}), 67.4 (C^2), 56.8 (C^9), 55.5 (C^{17}). ESI-LRMS *m/z* 414.29 ($[C_{21}H_{19}NO_8 + H]^+$, 100 %). ESI-HRMS calculated for $[C_{21}H_{20}NO_8]^+$ 414.1187 found 414.1189.

3. Metal binding studies with Mg^{2+} and Ca^{2+}

(a) Absorbance titration data for ligands not shown in the main text: CF_3-*p* and OMe-*p*; CF_3-*m* and OMe-*m*

The representative absorbance titration data and the associated fitting for these ligands are shown below in Figures S1 to S4. As in Figures 8 and 9 in the main text, the spectra shown in black are those of the metal-free ligands in each case. Spectra shown in purple and red are those recorded upon saturation with Mg^{2+} and Ca^{2+}, respectively. The titrations were performed in aqueous buffer solution (50 mM HEPES, pH 7.2, 0.1 M KCl) at 295 K with a ligand concentration of 25 μM.

(b) Fluorescence spectra of the free ligands and in the presence of saturating amounts of Mg^{2+} and Ca^{2+}

The emission spectra appear in Figures S5 and S6 below, conditions and legend as above. Figure S5 gives the raw spectra, showing the net change in intensity in each case, and Figure S6 shows the same spectra but intensity-normalized, to highlight any change in λ_{max}.

continues overpage

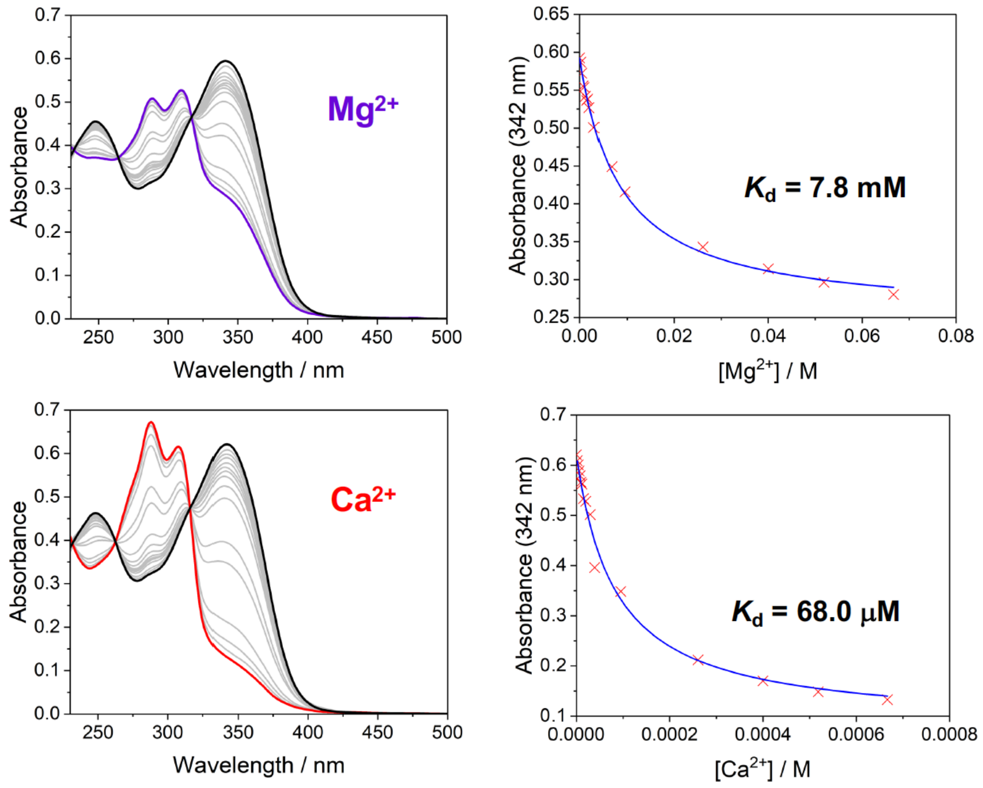

Figure S1. *Representative absorption spectral data (left) and associated fitting data (right) of the absorbance at 342 nm for the binding of* Mg^{2+} *(top) and* Ca^{2+} *(bottom) to* ***CF_3-p****;.*

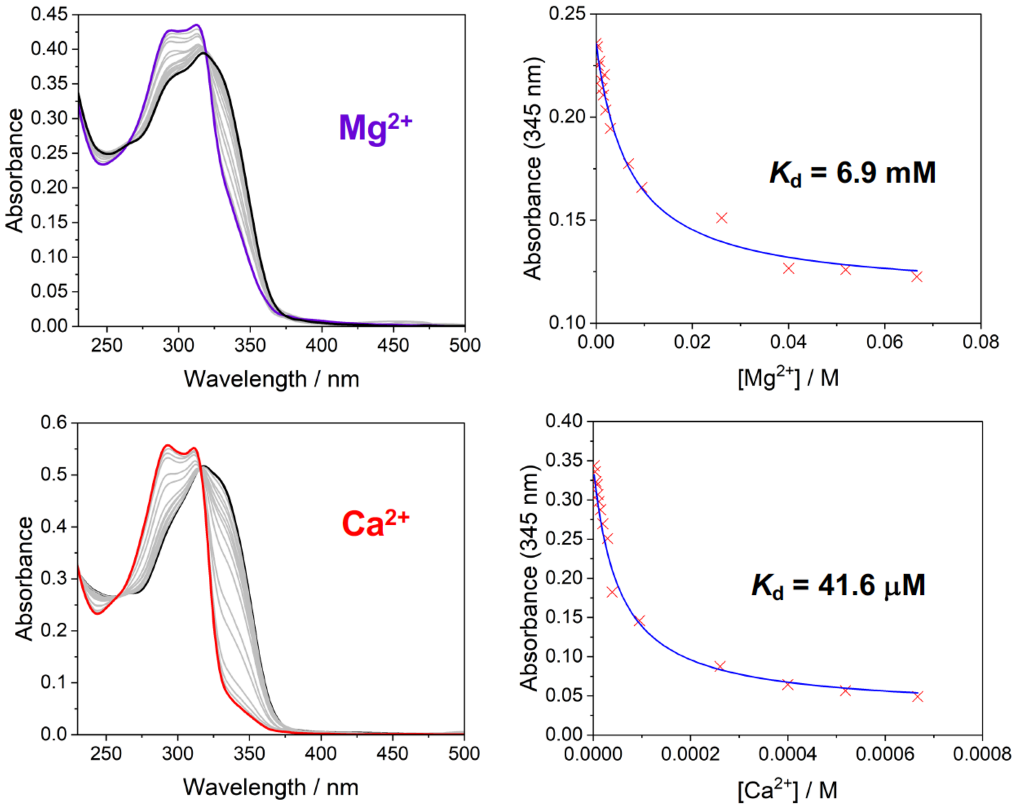

Figure S2. *Representative absorption spectral data (left) and associated fitting data (right) of the absorbance at 345 nm for the binding of* Mg^{2+} *(top) and* Ca^{2+} *(bottom) to* ***OMe-p****.*

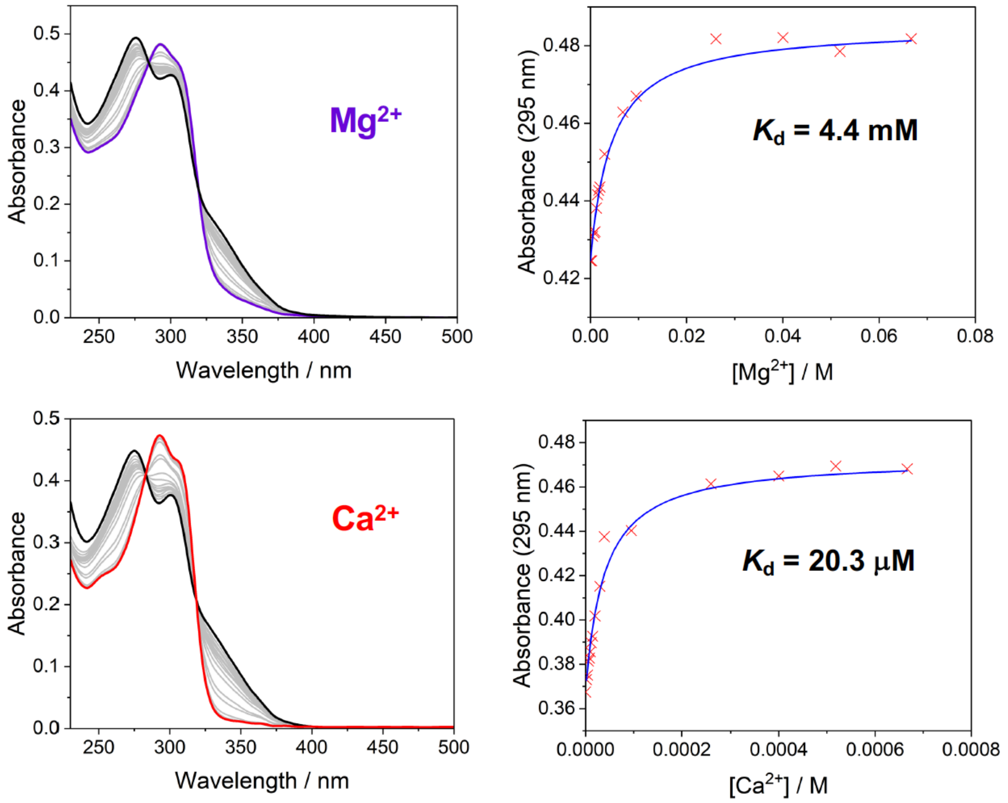

Figure S3. *Representative absorption spectral data (left) and associated fitting data (right) of the absorbance at 295 nm for the binding of* Mg^{2+} *(top) and* Ca^{2+} *(bottom) to* ***CF3-m****.*

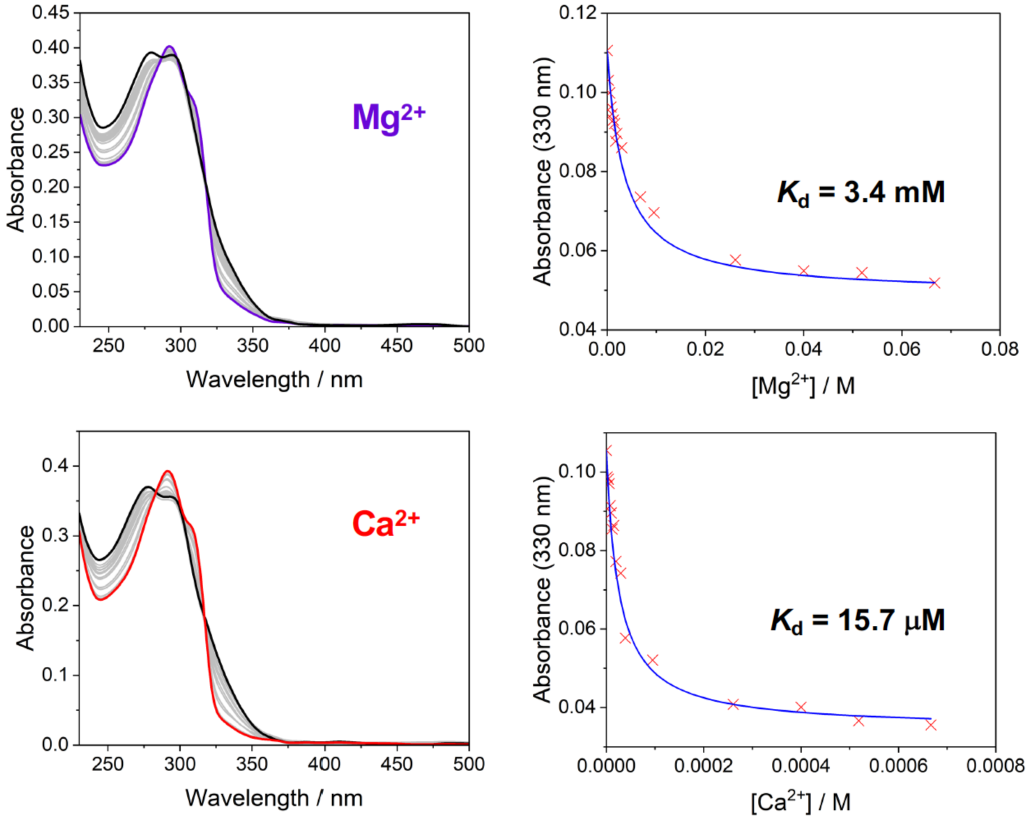

Figure S4. *Representative absorption spectral data (left) and associated fitting data (right) of the absorbance at 330 nm for the binding of* Mg^{2+} *(top) and* Ca^{2+} *(bottom) to* ***OMe-m****.*

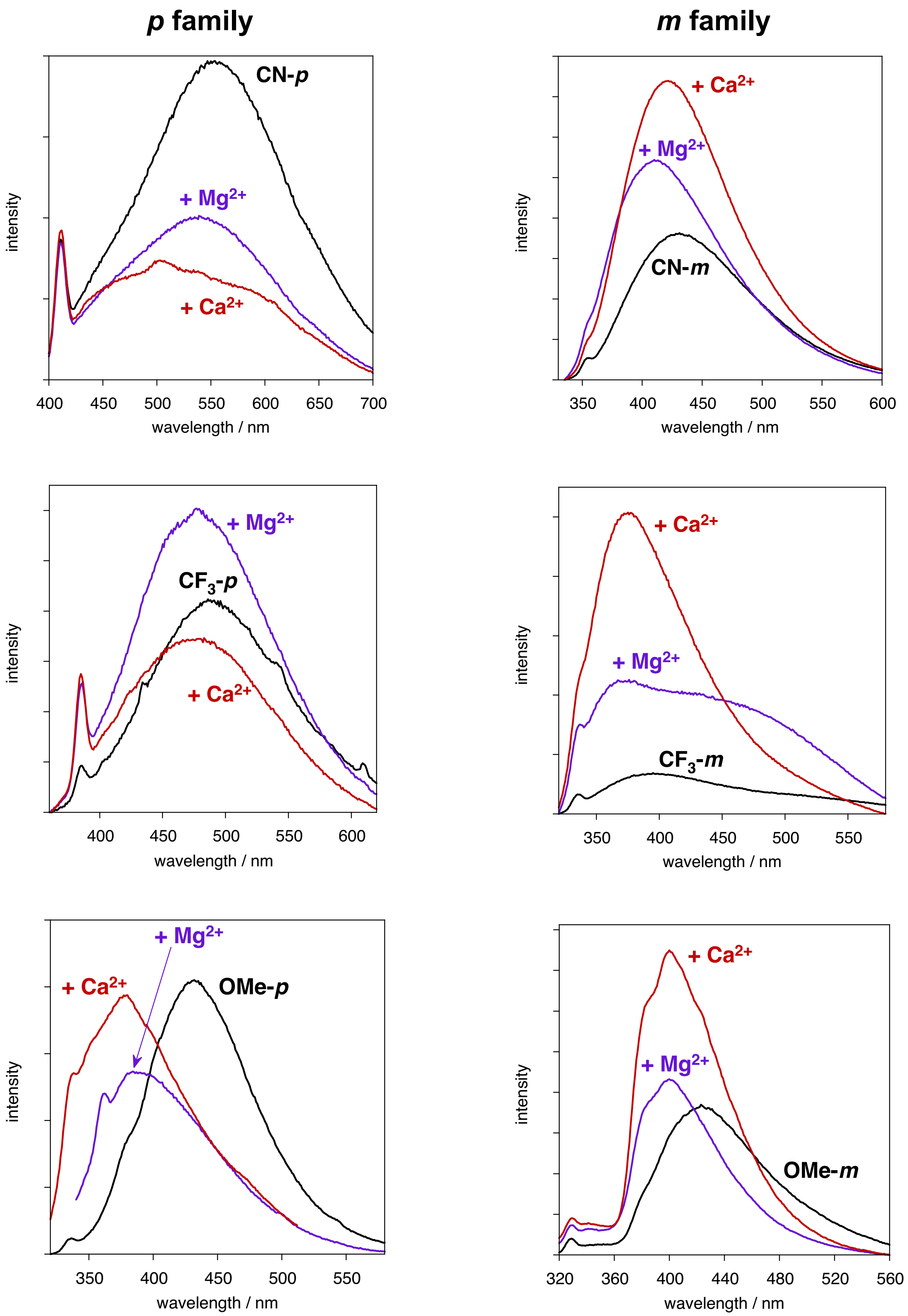

Figure S5. *Fluorescence spectra of the free ligands (black lines) and at the end points of the absorption titrations with Mg^{2+} (purple) or Ca^{2+} (red) shown in Figures 8, 9, and S1-4. Samples were excited into the lowest-energy absorption bands; the sharp peaks to high-energy of the fluorescence bands are the O–H Raman bands of water.*

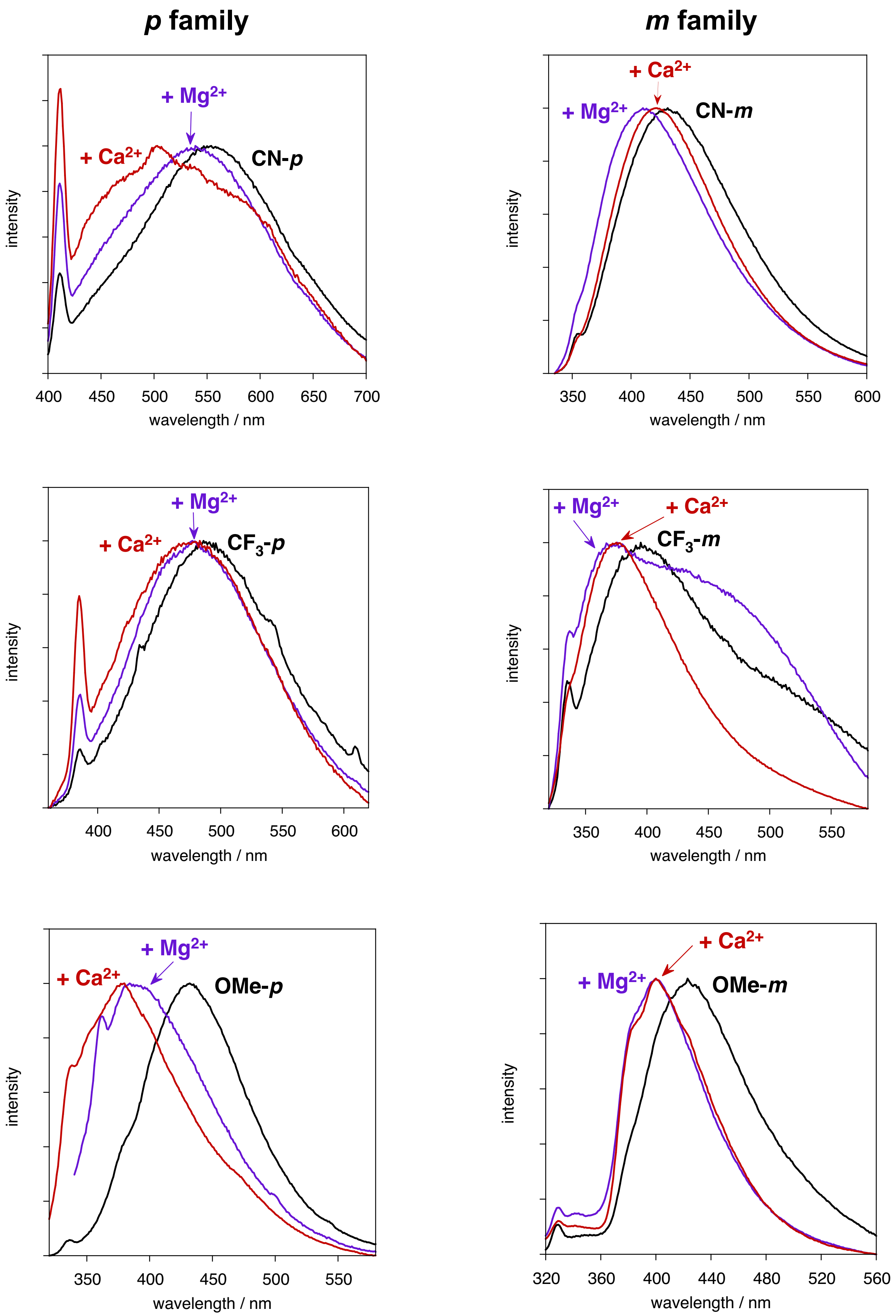

Figure S6. *Fluorescence spectra as in Figure S5 but shown here intensity-normalized.*

4. NMR spectra of the ester precursors and the final carboxylate ligands

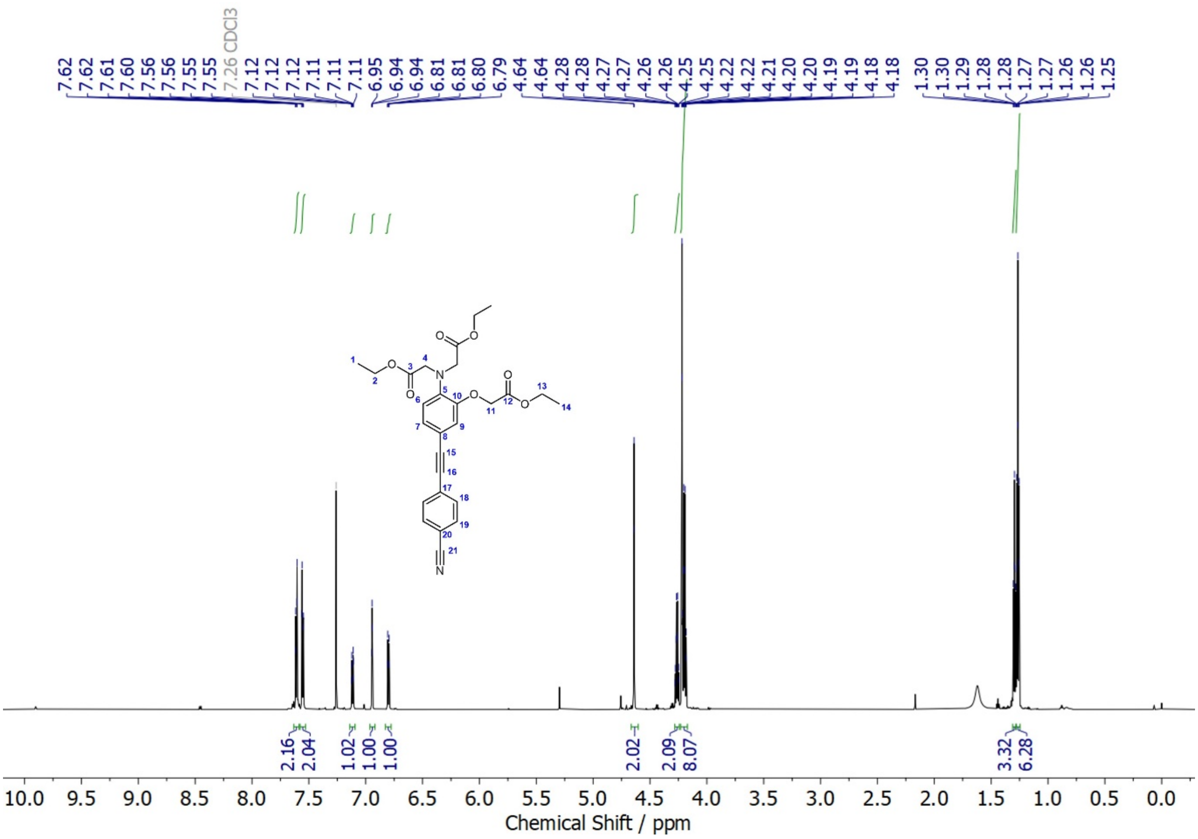

Figure S7. [1]*H NMR spectrum of* ***CN-p-Et₃*** *in* $CDCl_3$*.*

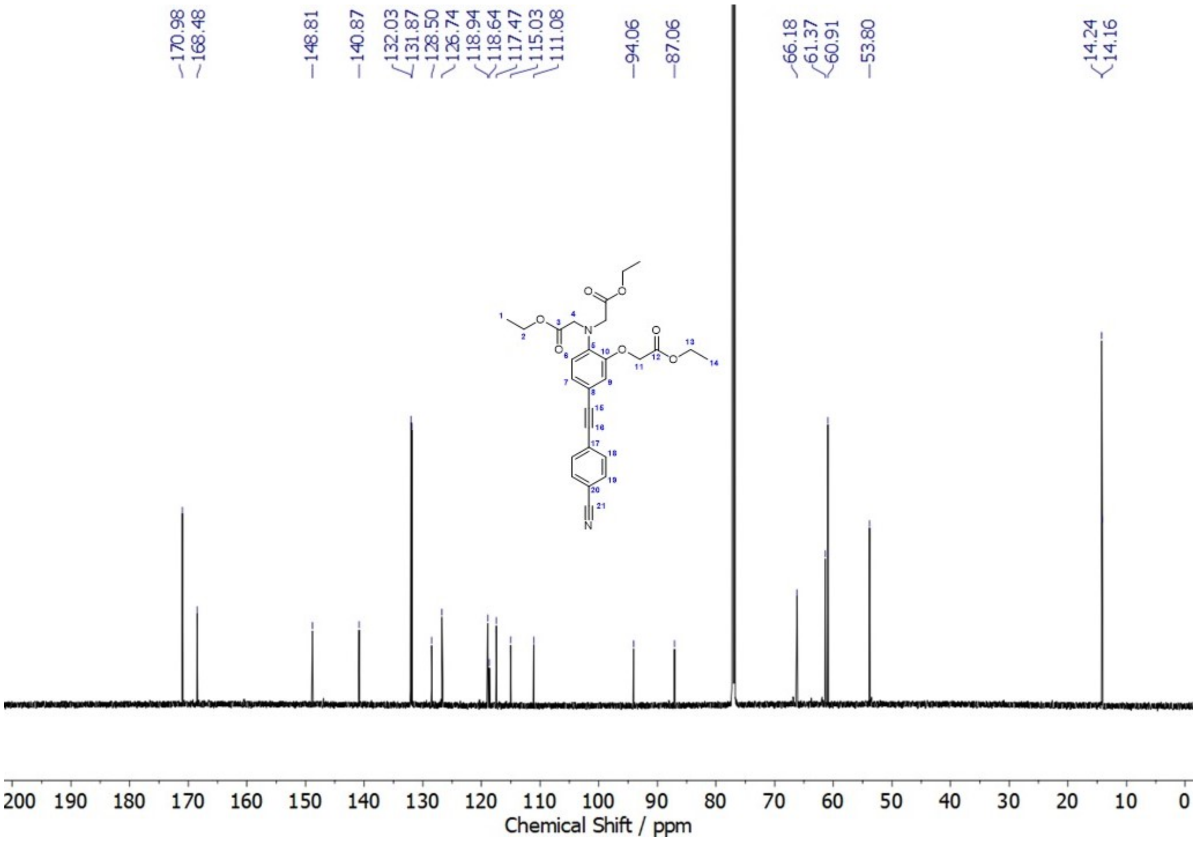

Figure S8. [13]*C NMR spectrum of* ***CN-p-Et₃*** *in* $CDCl_3$*.*

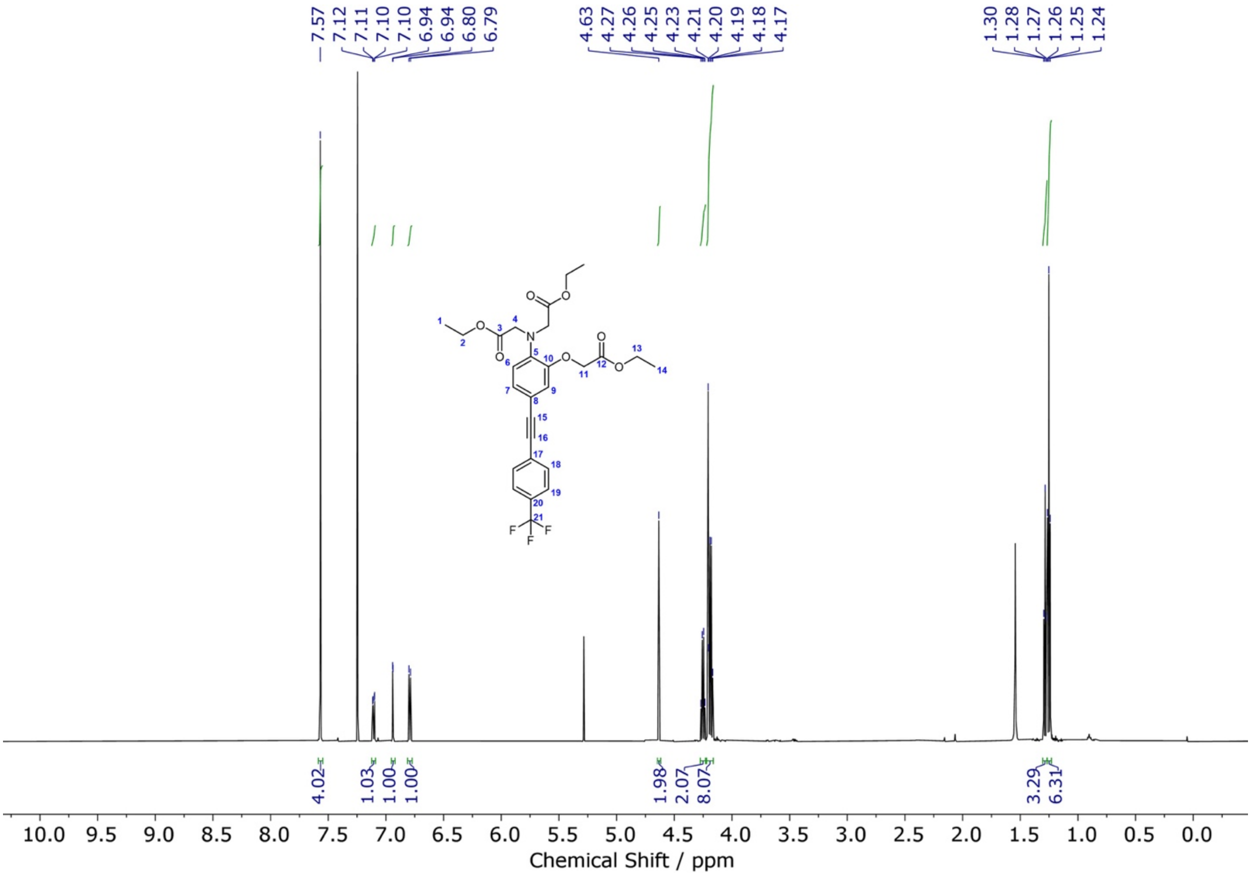

Figure S9. *^{1}H NMR spectrum of **CF_3-p-Et_3** in $CDCl_3$.*

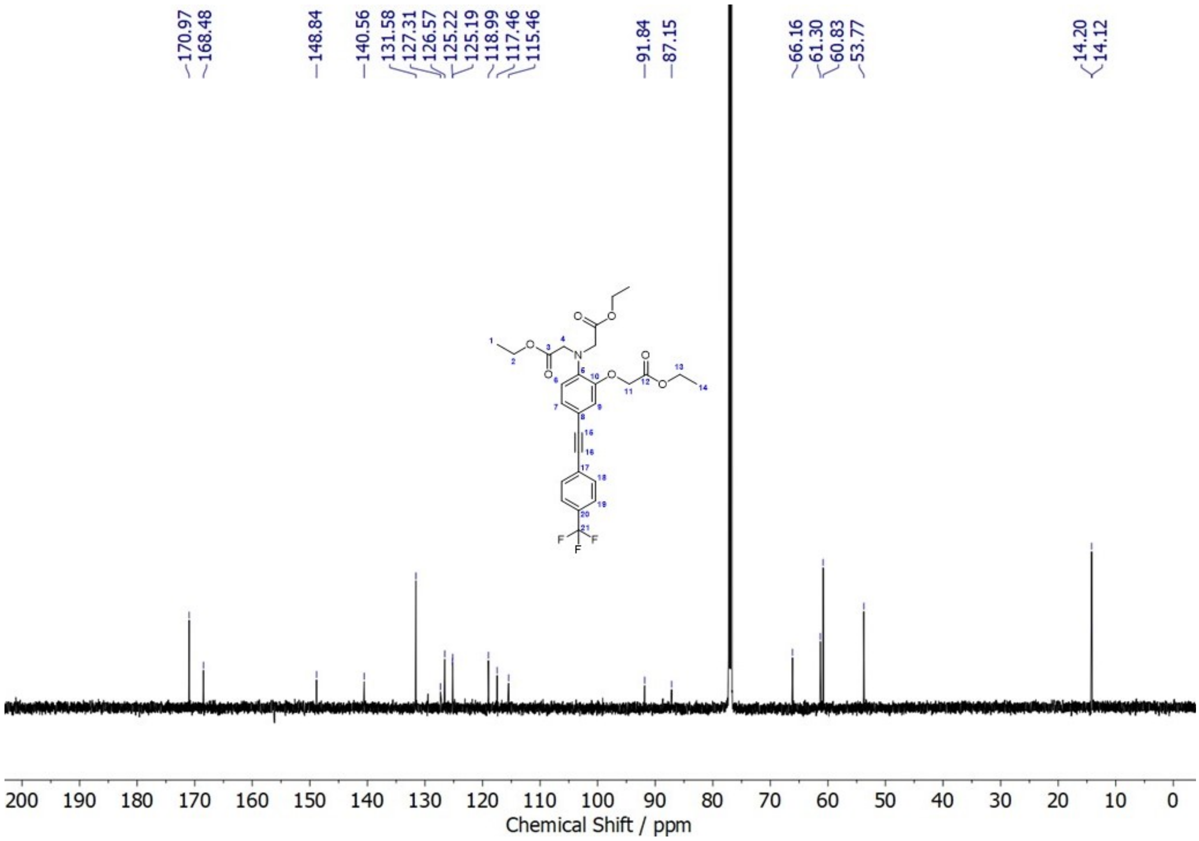

Figure S10. *^{13}C NMR spectrum of **CF_3-p-Et_3** in $CDCl_3$.*

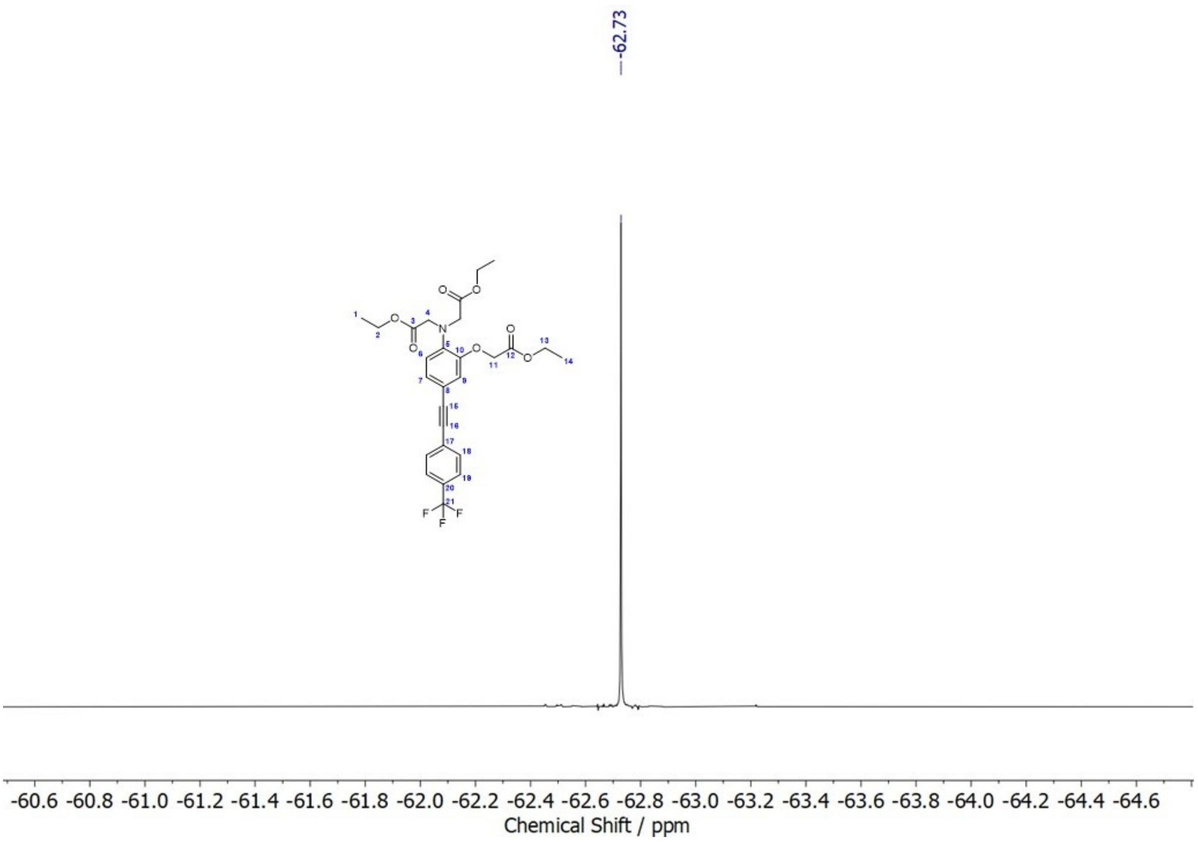

Figure S11. *^{19}F NMR spectrum of* ***CF₃-p-Et₃*** *in CDCl₃.*

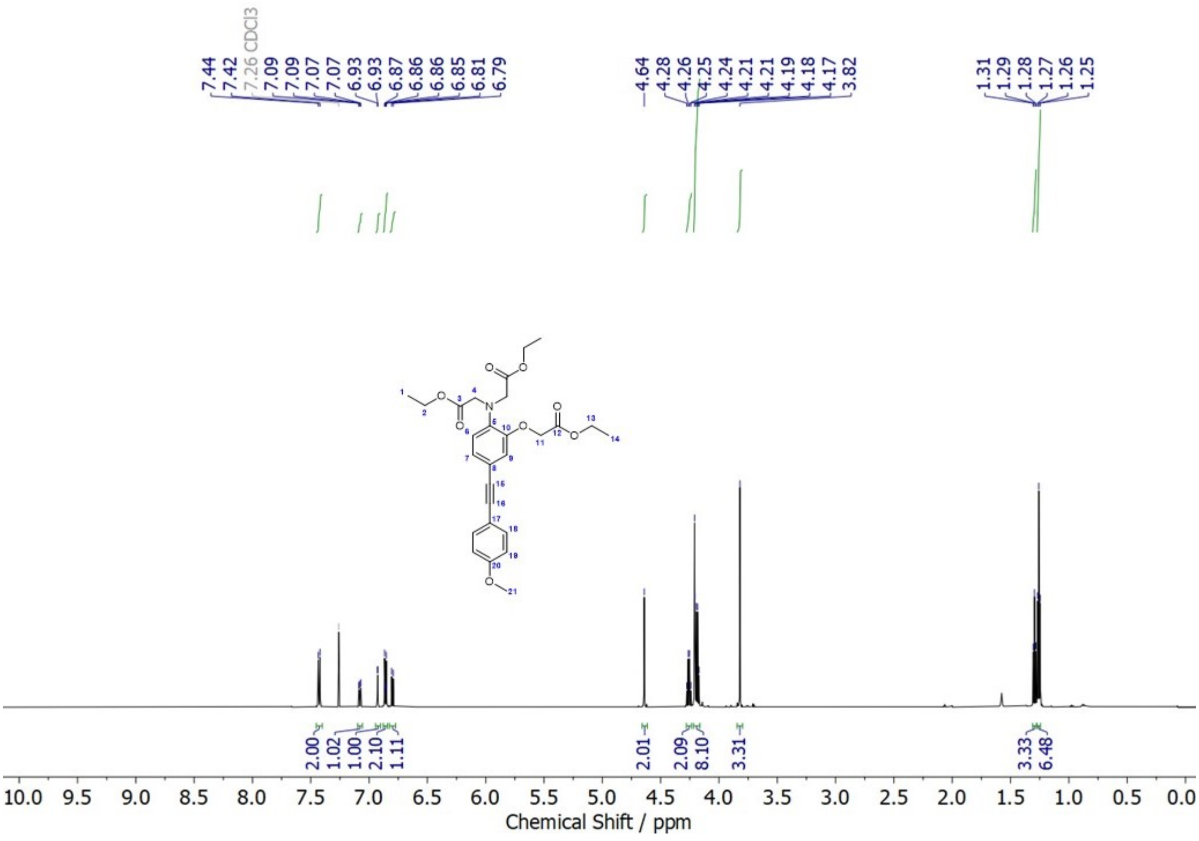

Figure S12. *^{1}H NMR spectrum of* ***OMe-p-Et₃*** *in CDCl₃.*

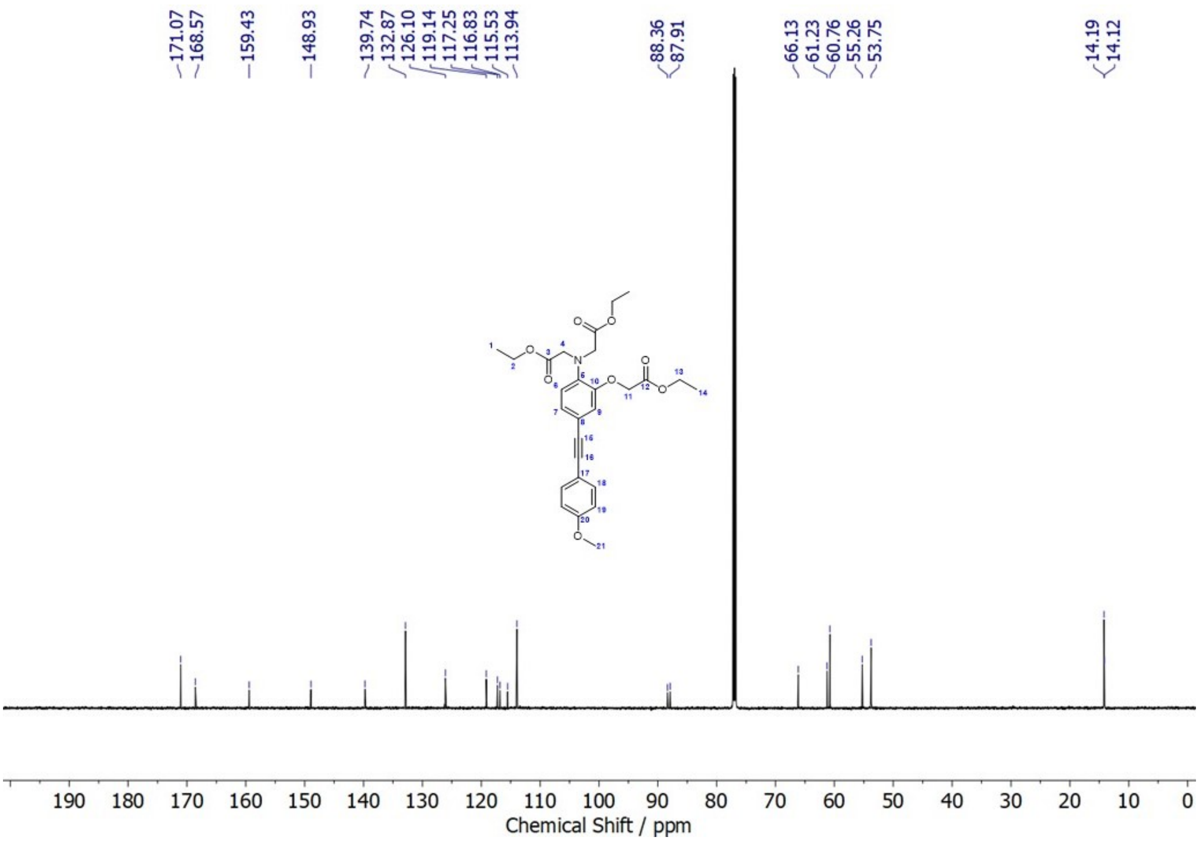

Figure S13. *^{13}C NMR spectrum of* ***OMe-p-Et₃*** *in CDCl₃.*

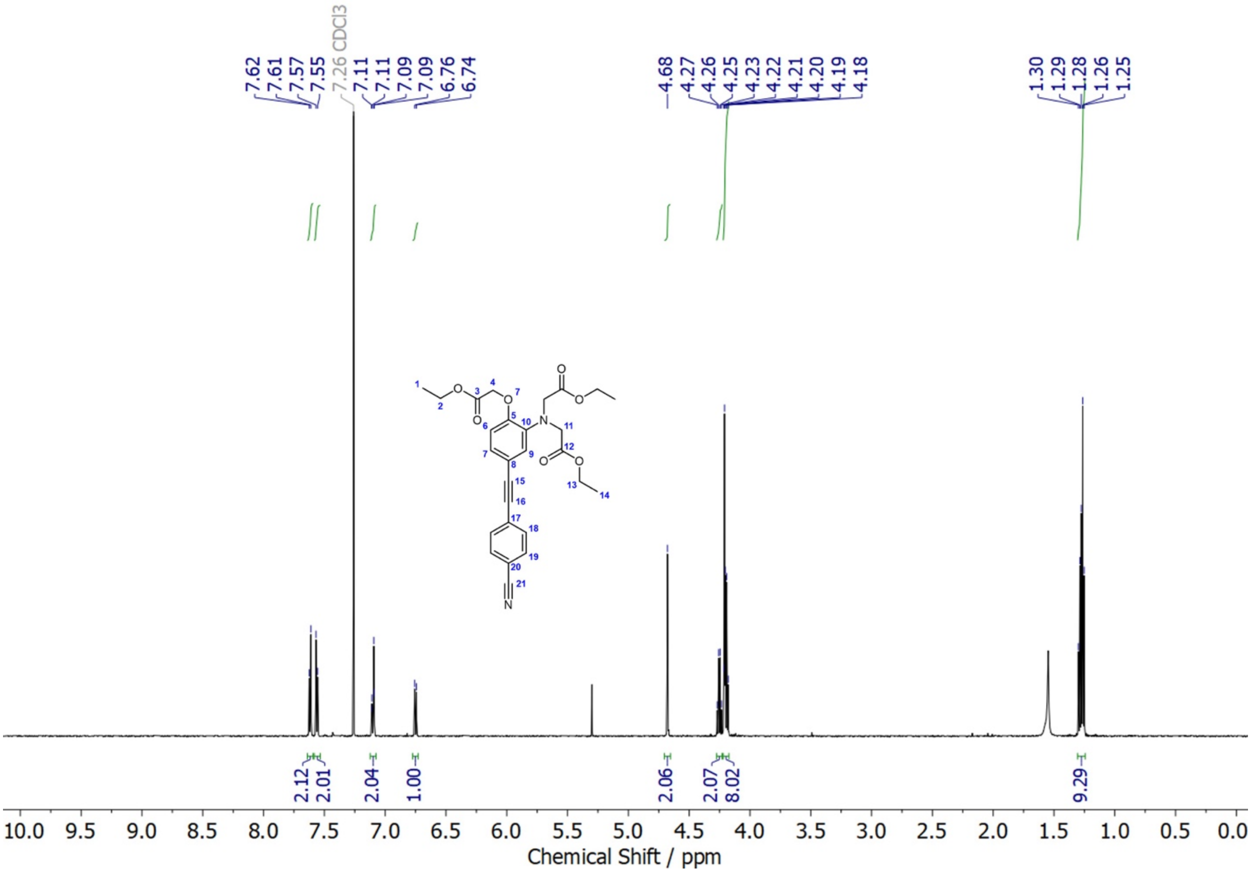

Figure S14. *^{1}H NMR spectrum of* ***CN-m-Et₃*** *in CDCl₃.*

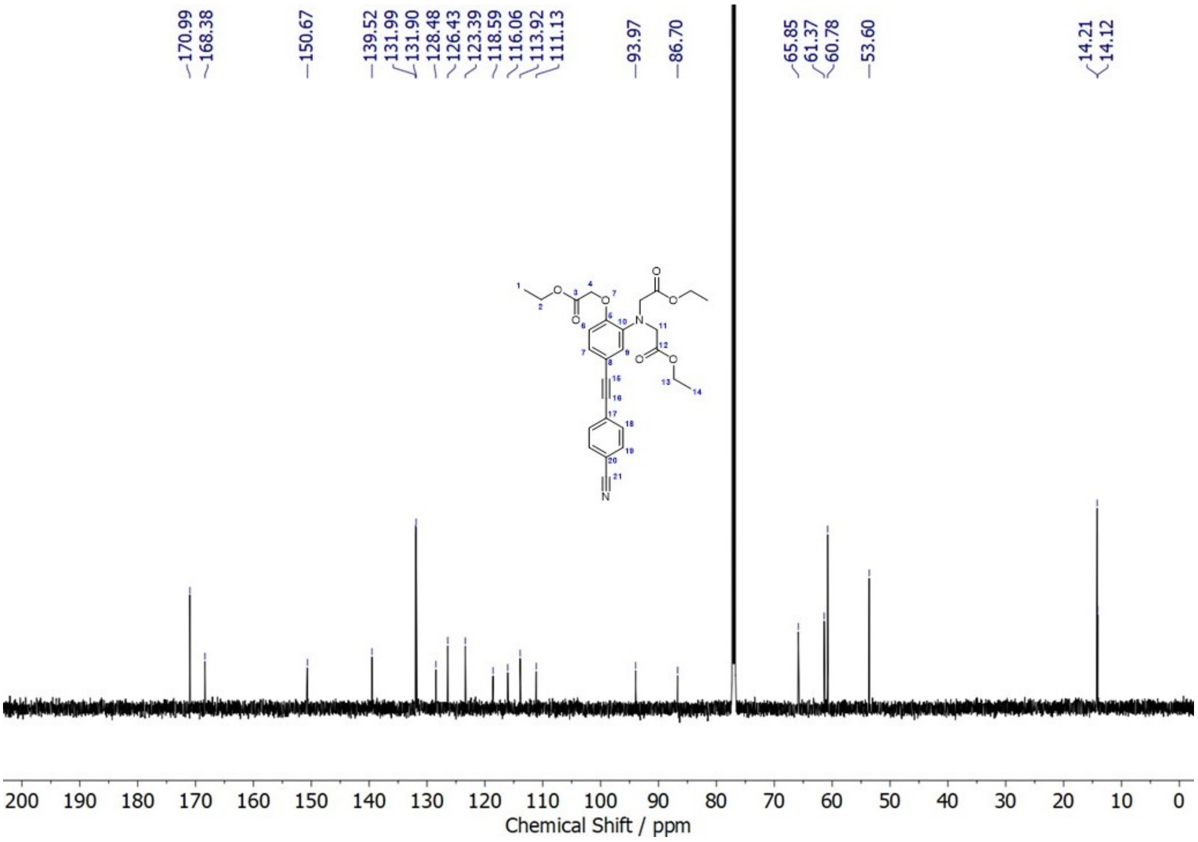

Figure S15. *^{13}C NMR Spectrum of **CN-m-Et$_3$** in CDCl$_3$.*

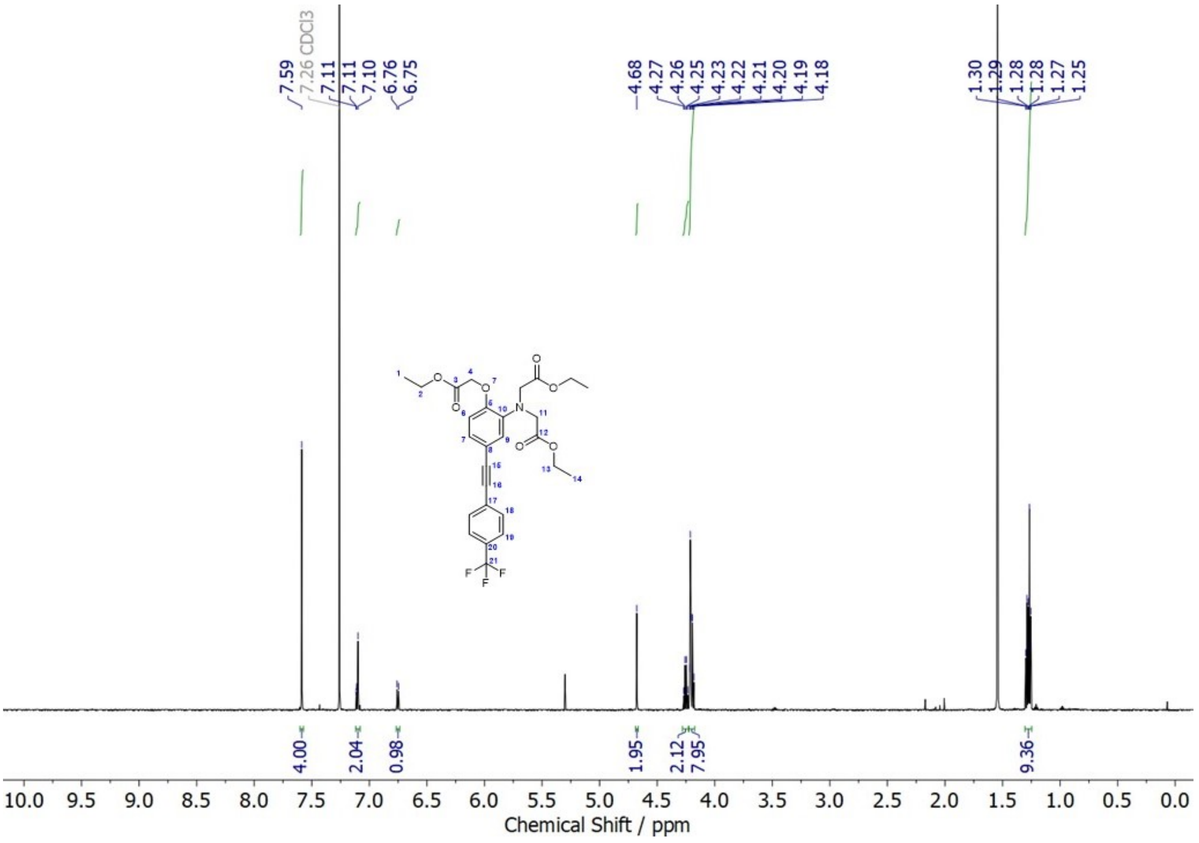

Figure S16. *^{1}H NMR Spectrum of **CF$_3$-m-Et$_3$** in CDCl$_3$.*

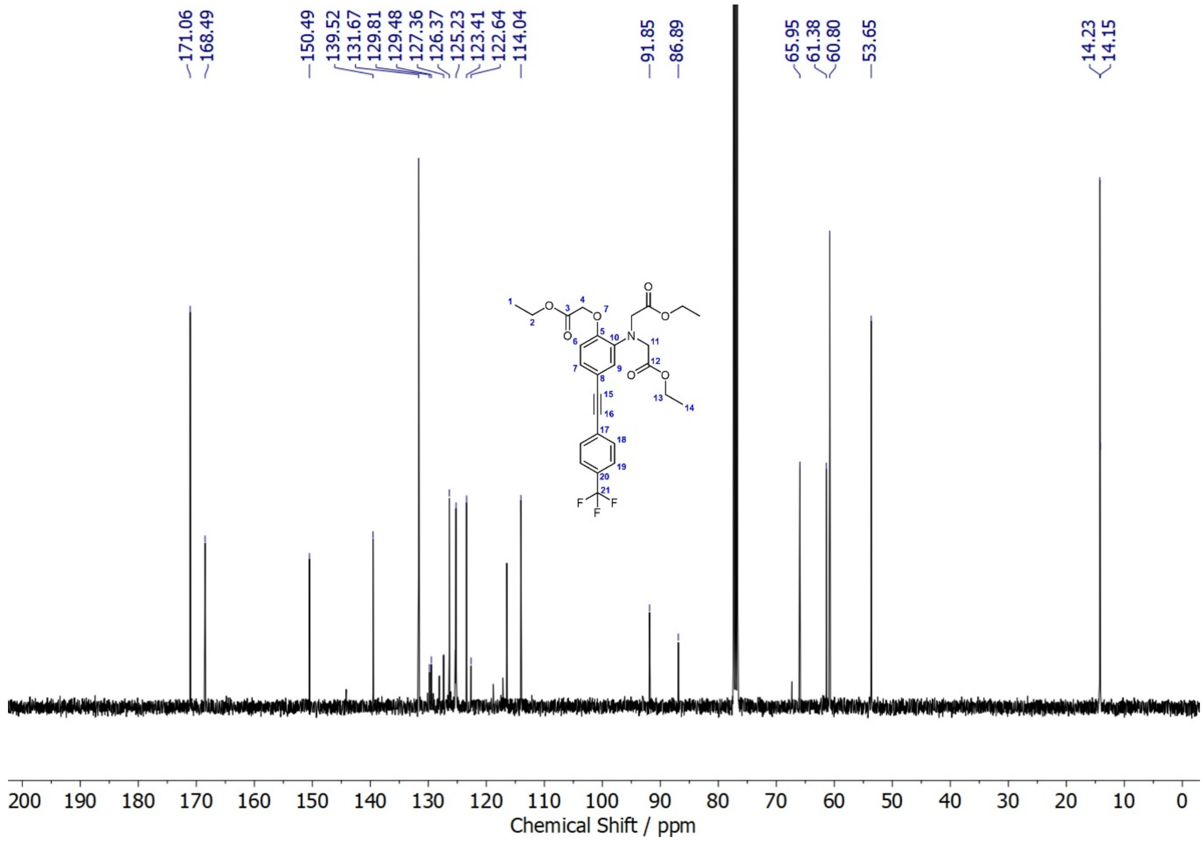

Figure S17. *^{13}C NMR Spectrum of* ***CF_3-m-Et_3*** *in $CDCl_3$.*

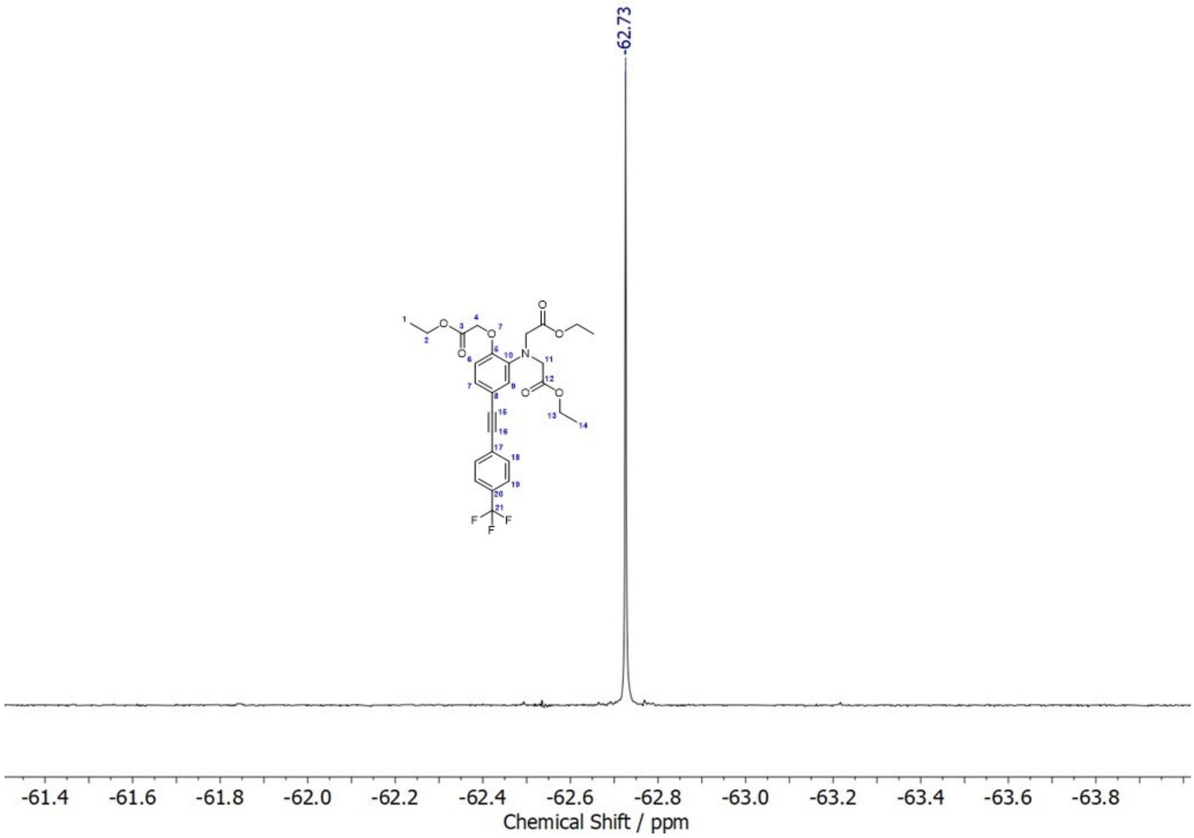

Figure S18. *^{19}F NMR Spectrum of* ***CF_3-m-Et_3*** *in $CDCl_3$.*

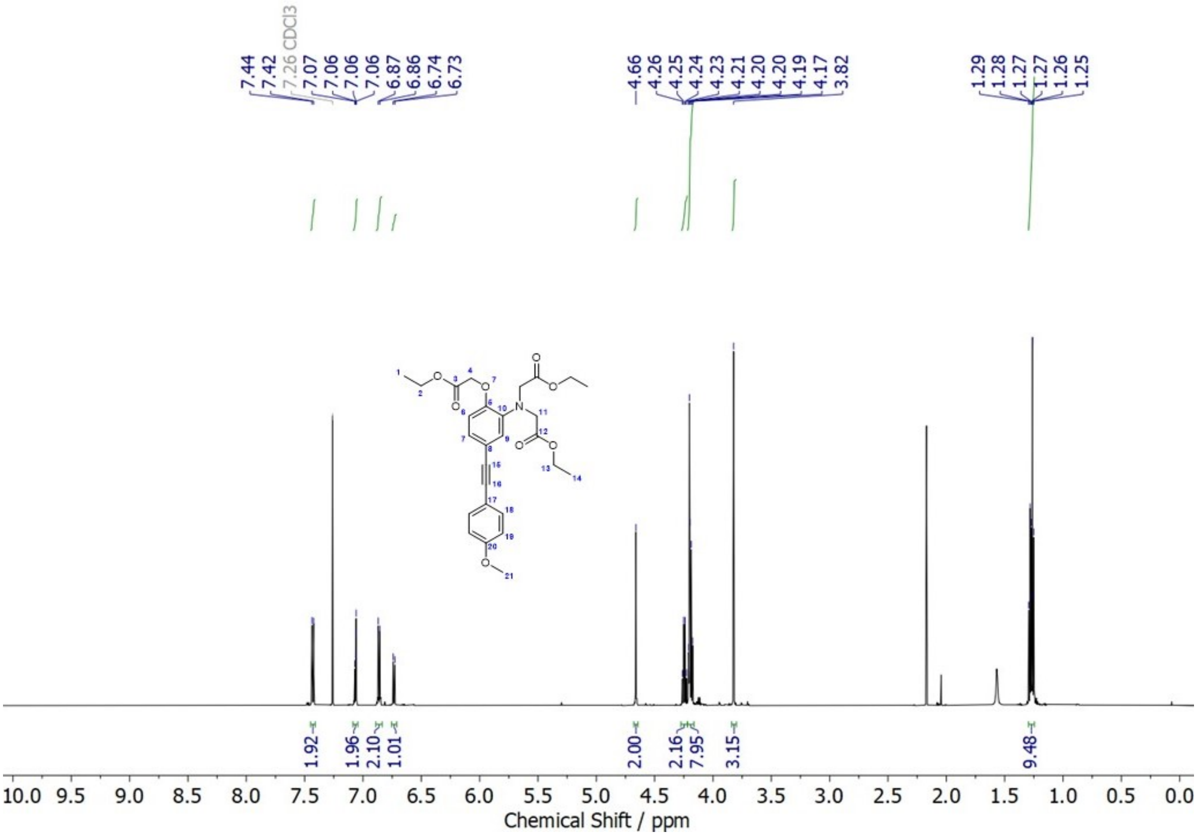

Figure S19. *^{1}H NMR Spectrum of* ***OMe-m-Et$_3$*** *in CDCl$_3$.*

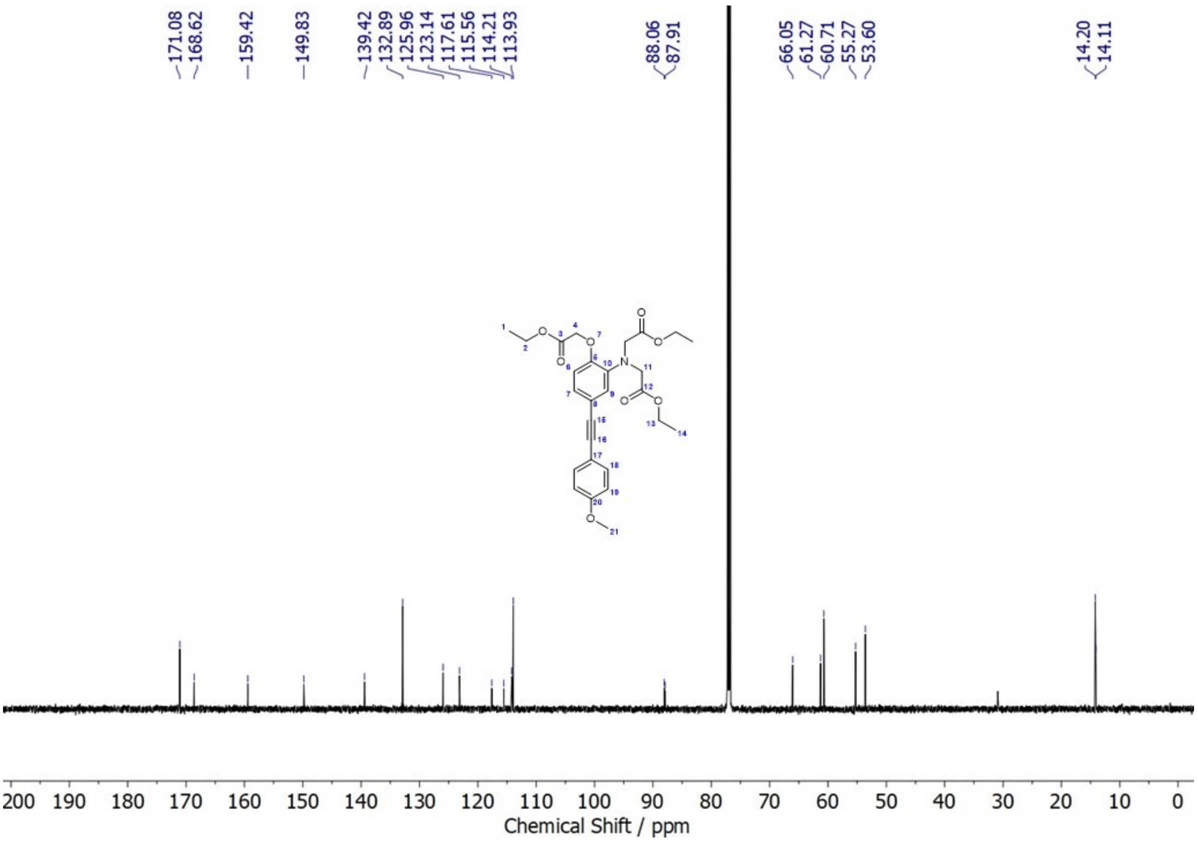

Figure S20. *^{13}C NMR Spectrum of* ***OMe-m-Et$_3$*** *in CDCl$_3$.*

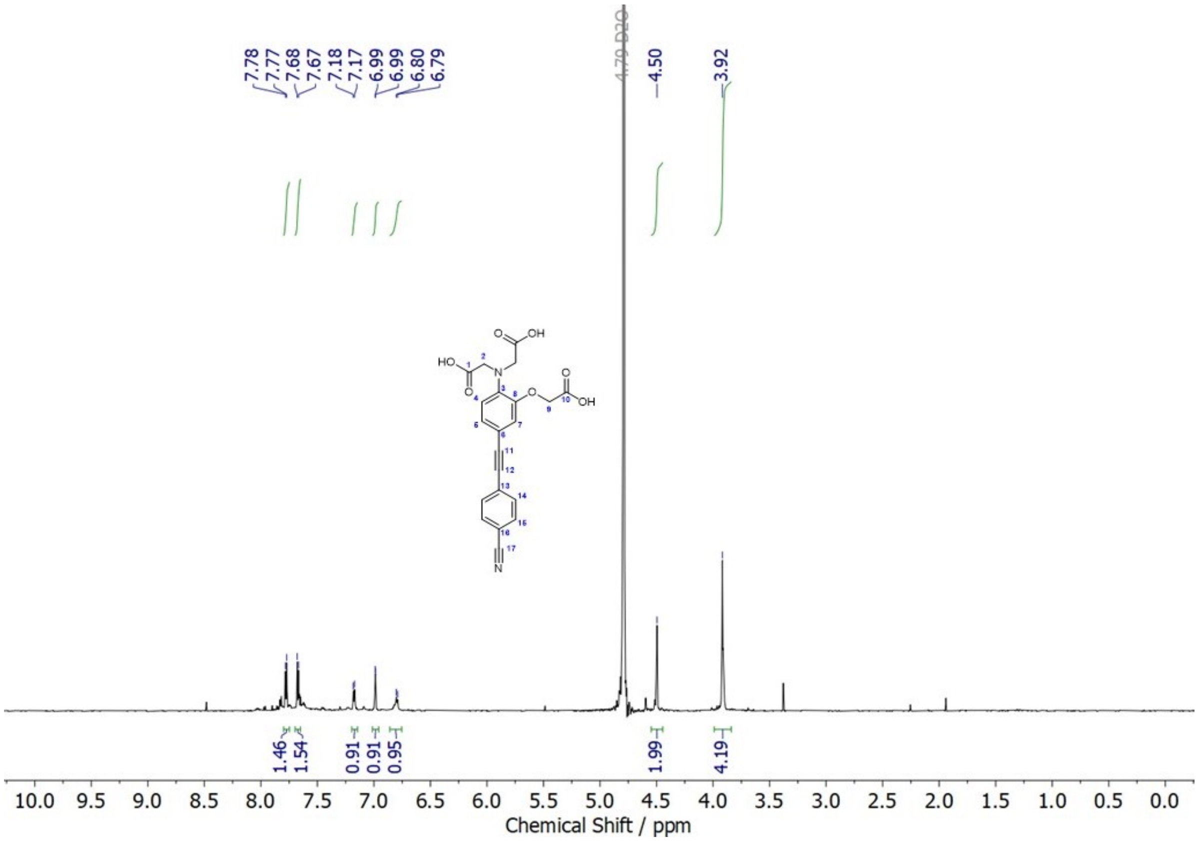

Figure S21. *^{1}H NMR Spectrum of* ***CN-p*** *in* D_2O.

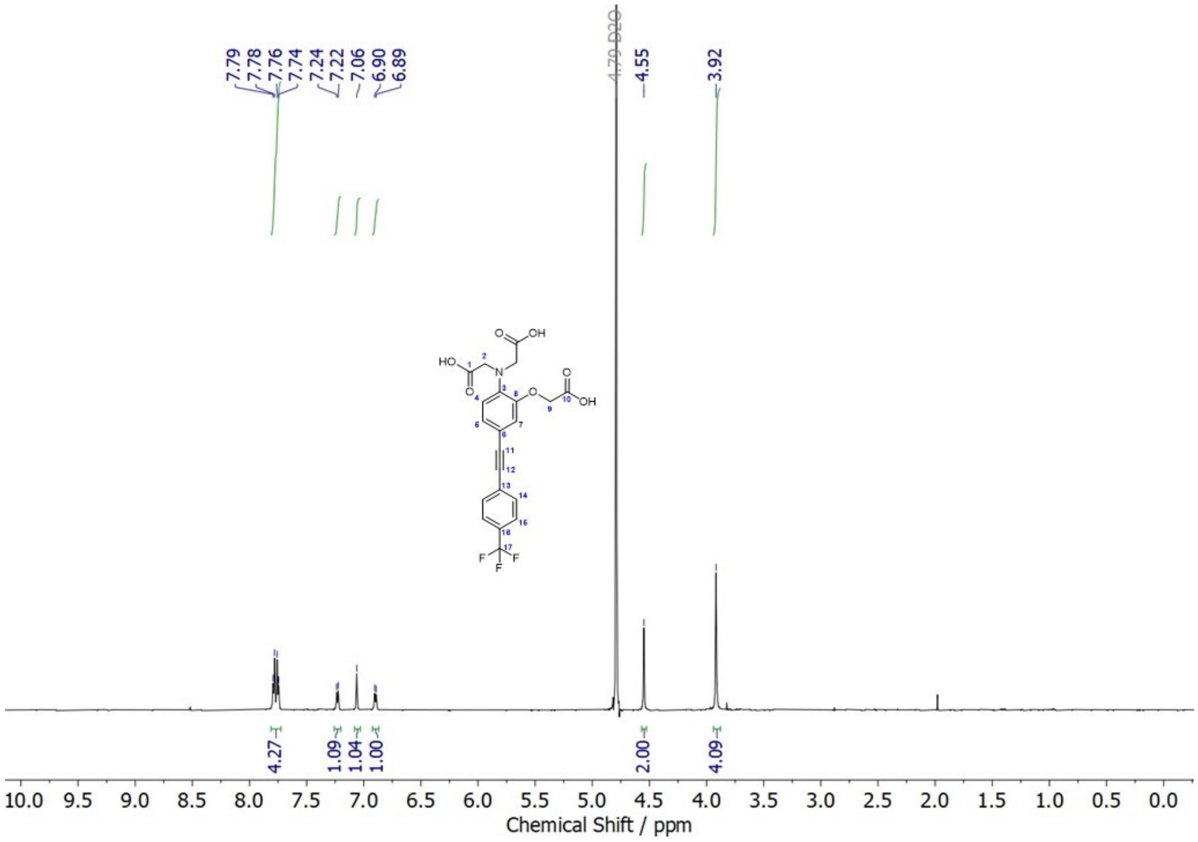

Figure S22. *^{1}H NMR Spectrum of* ***CF_3-p*** *in* D_2O.

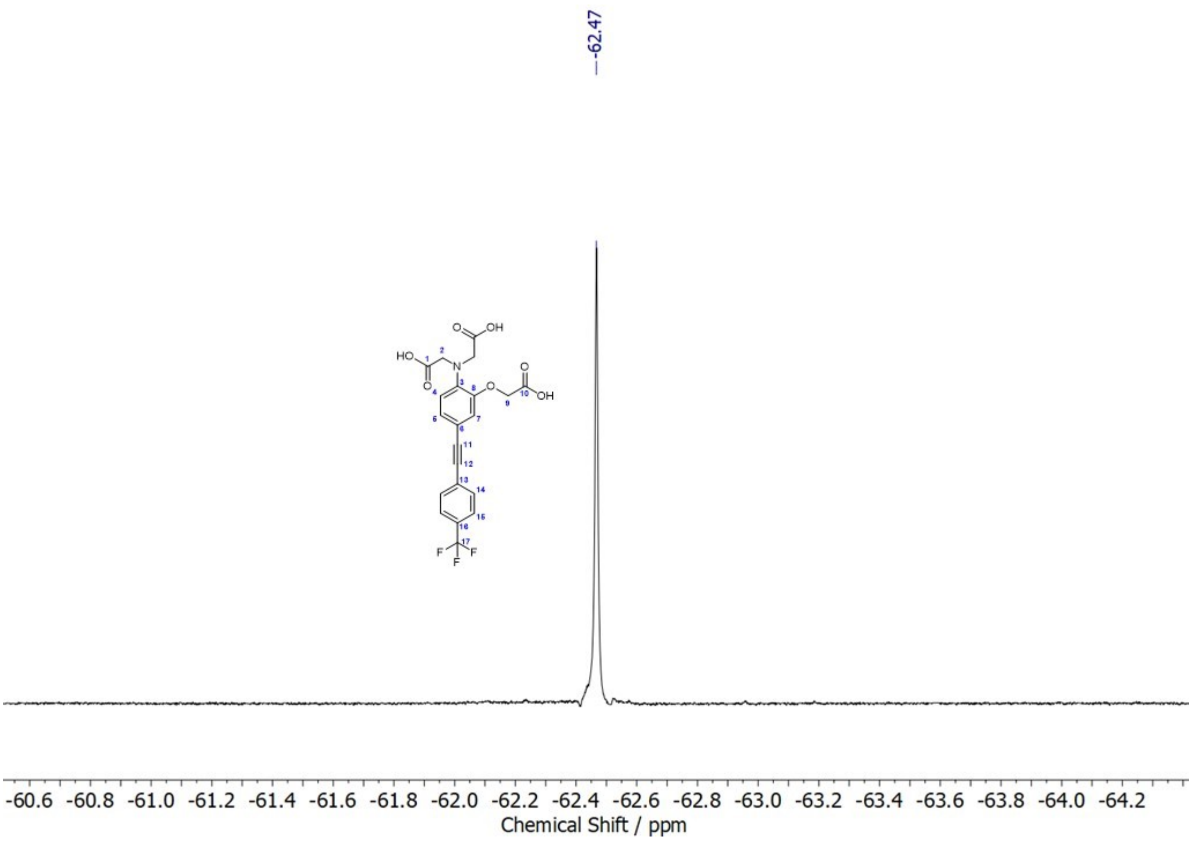

Figure S23. *^{19}F NMR Spectrum of* ***CF_3-p*** *in D_2O.*

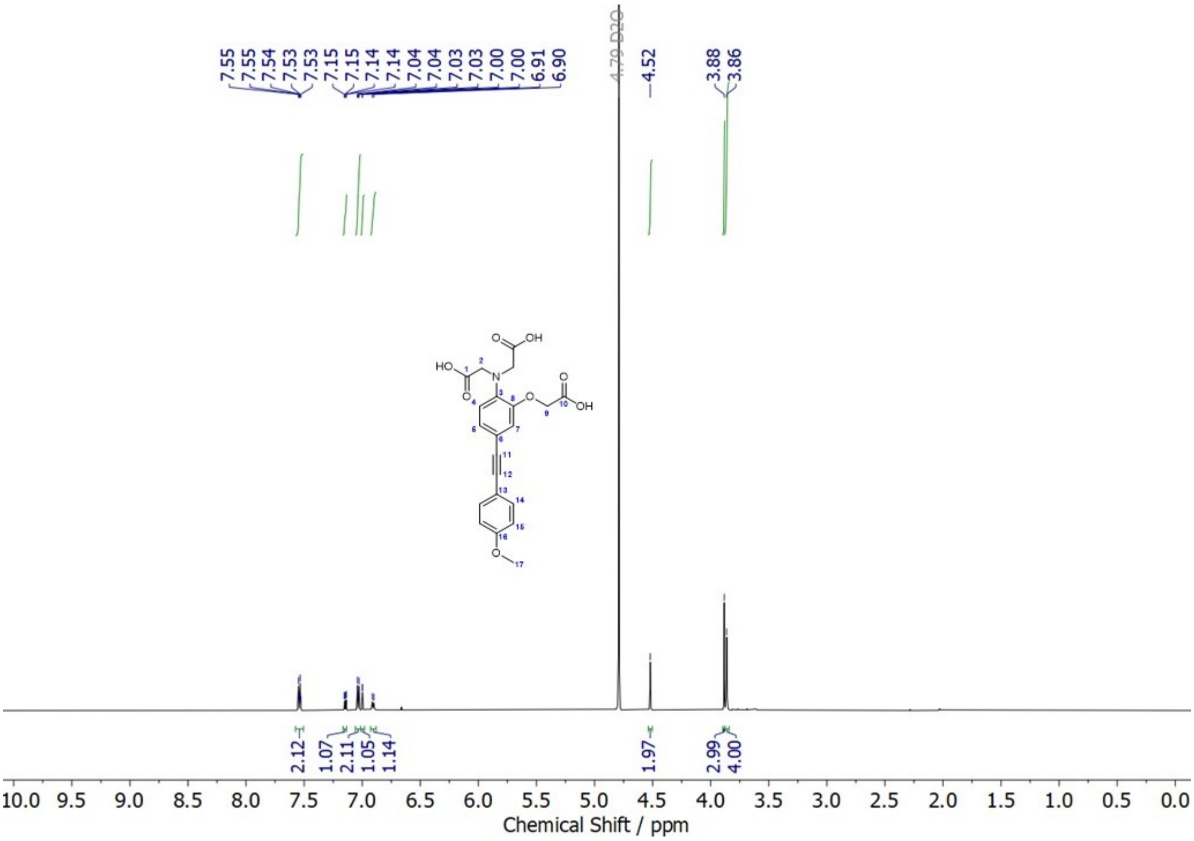

Figure S24. *^{1}H NMR Spectrum of* ***OMe-p*** *in D_2O.*

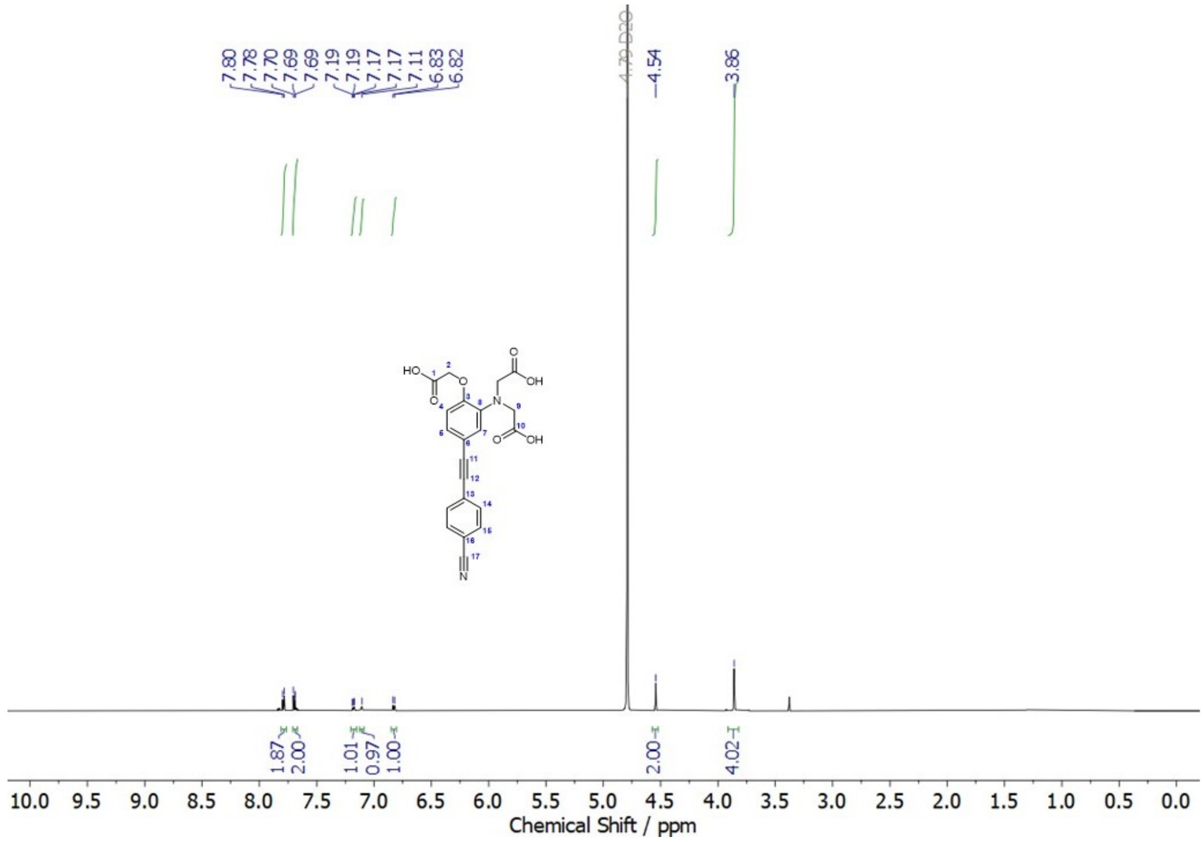

Figure S25. *^{1}H NMR Spectrum of* ***CN-m*** *in D_2O.*

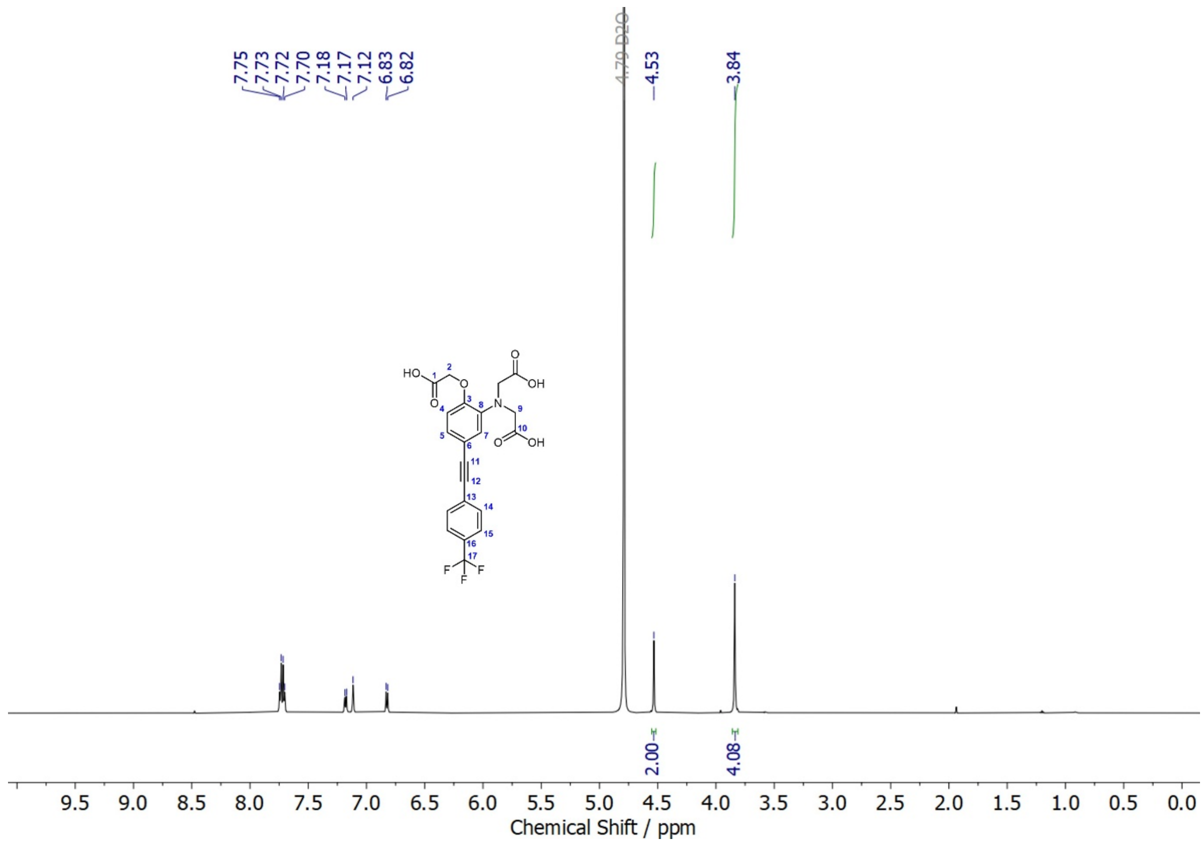

Figure S26. *^{1}H NMR Spectrum of* ***CF_3-m*** *in D_2O.*

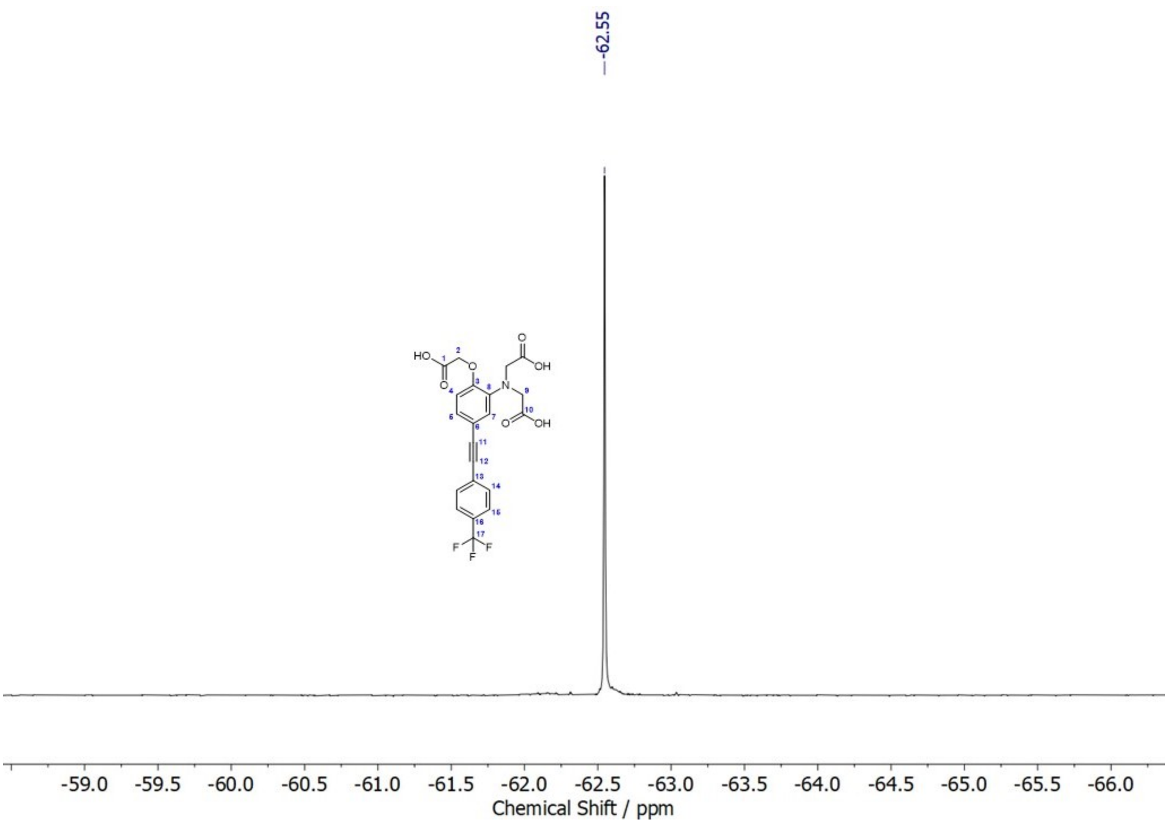

Figure S27. *^{19}F NMR Spectrum of* ***CF_3-m*** *in D_2O.*

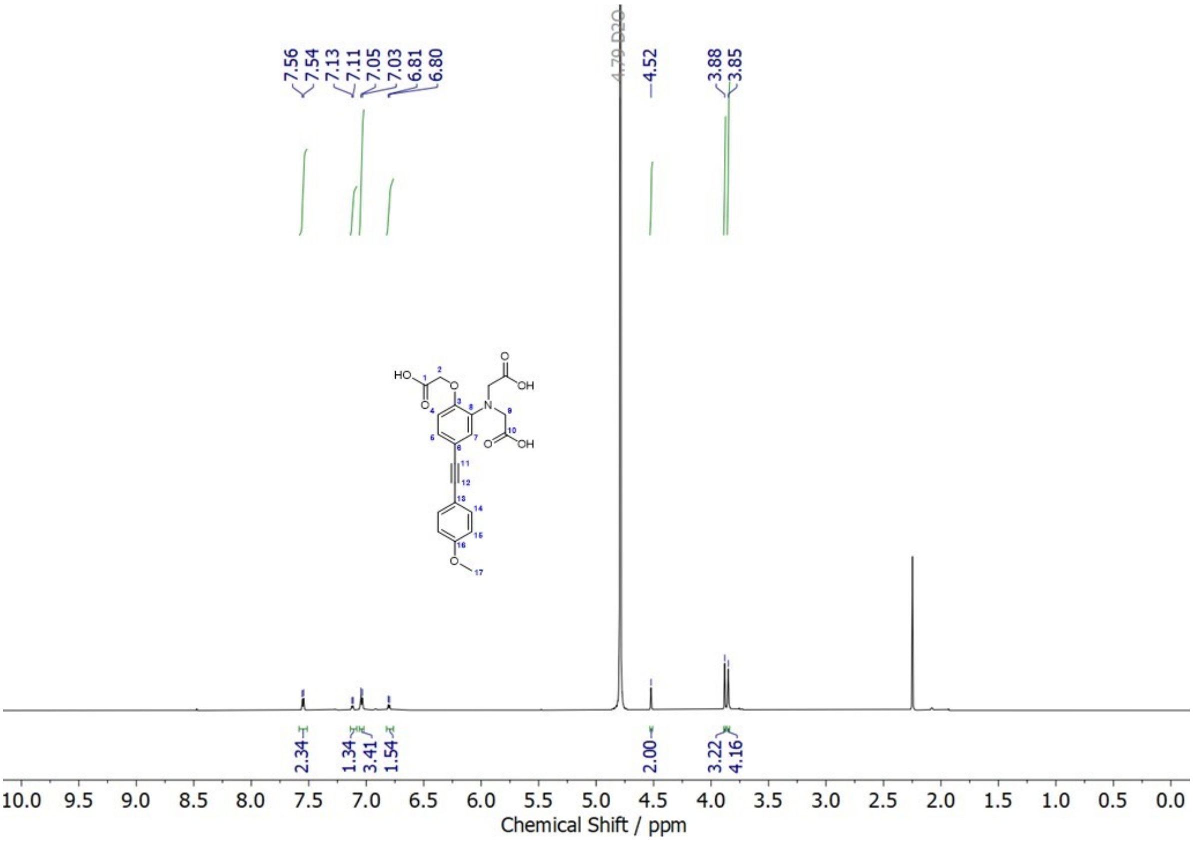

Figure S28. *^{1}H NMR Spectrum of* ***OMe-m*** *in D_2O.*
